# Metabolomic Evidence for Peroxisomal Dysfunction in Myalgic Encephalomyelitis/Chronic Fatigue Syndrome

**DOI:** 10.3390/ijms23147906

**Published:** 2022-07-18

**Authors:** Xiaoyu Che, Christopher R. Brydges, Yuanzhi Yu, Adam Price, Shreyas Joshi, Ayan Roy, Bohyun Lee, Dinesh K. Barupal, Aaron Cheng, Dana March Palmer, Susan Levine, Daniel L. Peterson, Suzanne D. Vernon, Lucinda Bateman, Mady Hornig, Jose G. Montoya, Anthony L. Komaroff, Oliver Fiehn, W. Ian Lipkin

**Affiliations:** 1 Center for Infection and Immunity, Mailman School of Public Health, Columbia University, New York, NY 10032, USA; xc2273@cumc.columbia.edu (X.C.); price0416@gmail.com (A.P.); sj3038@cumc.columbia.edu (S.J.); ar4239@cumc.columbia.edu (A.R.); kh2907@columbia.edu (B.L.); aaron.cheng@caa.columbia.edu (A.C.); 2 Department of Biostatistics, Mailman School of Public Health, Columbia University, New York, NY 10032, USA; yy3019@cumc.columbia.edu; 3 UC Davis Genome Center—Metabolomics, University of California, Davis, CA 95616, USA; crbrydges@ucdavis.edu; 4 Department of Environmental Medicine and Public Health, Icahn School of Medicine at Mount Sinai, New York, NY 10029, USA; dinesh.kumar@mssm.edu; 5 Department of Epidemiology, Mailman School of Public Health, Columbia University, New York, NY 10032, USA; dm2025@columbia.edu (D.M.P.); mh2092@cumc.columbia.edu (M.H.); 6 Levine Clinic, New York, NY 10021, USA; susanmlevinemd@gmail.com; 7 Sierra Internal Medicine at Incline Village, Incline Village, NV 89451, USA; dpeterson@sierrainternalmed.com; 8 Bateman Horne Center, Salt Lake City, UT 84102, USA; sdvernon@batemanhornecenter.org (S.D.V.); lbateman@batemanhornecenter.org (L.B.); 9 Sutter Health Palo Alto Medical Foundation, Palo Alto, CA 94301, USA; montoyj@sutterhealth.org; 10 Harvard Medical School, Brigham and Women’s Hospital, Boston, MA 02115, USA; anthony_komaroff@hms.harvard.edu

**Keywords:** myalgic encephalomyelitis, chronic fatigue syndrome, metabolomics, biomarker, peroxisome, cytidine-5′-diphosphocholine pathway, tricarboxylic acid cycle

## Abstract

Myalgic encephalomyelitis/chronic fatigue syndrome (ME/CFS) is a chronic and debilitating disease characterized by unexplained physical fatigue, cognitive and sensory dysfunction, sleeping disturbances, orthostatic intolerance, and gastrointestinal problems. People with ME/CFS often report a prodrome consistent with infections. Using regression, Bayesian and enrichment analyses, we conducted targeted and untargeted metabolomic analysis of plasma from 106 ME/CFS cases and 91 frequency-matched healthy controls. Subjects in the ME/CFS group had significantly decreased levels of plasmalogens and phospholipid ethers (*p* < 0.001), phosphatidylcholines (*p* < 0.001) and sphingomyelins (*p* < 0.001), and elevated levels of dicarboxylic acids (*p* = 0.013). Using machine learning algorithms, we were able to differentiate ME/CFS or subgroups of ME/CFS from controls with area under the receiver operating characteristic curve (AUC) values up to 0.873. Our findings provide the first metabolomic evidence of peroxisomal dysfunction, and are consistent with dysregulation of lipid remodeling and the tricarboxylic acid cycle. These findings, if validated in other cohorts, could provide new insights into the pathogenesis of ME/CFS and highlight the potential use of the plasma metabolome as a source of biomarkers for the disease.

## 1. Introduction

Myalgic encephalomyelitis/chronic fatigue syndrome (ME/CFS) is a disease of unknown cause that is defined by impairment from fatigue lasting longer than six months, unrefreshing sleep, post-exertional malaise, and either cognitive dysfunction or orthostatic intolerance [1]. People with ME/CFS may also report gastrointestinal disturbances, influenza-like symptoms, and chronic pain [2]. It is estimated that ME/CFS affects between 0.4% to 2.5% of the global population, and 1.5 to 2.5 million people in the United States alone [1,3]. There are no approved laboratory tests for ME/CFS. A diagnosis is based on medical history and the exclusion of other disorders that may result in chronic illness [4,5].

Prior metabolomic studies of patients with ME/CFS have provided insights into the potential pathogenesis and course of the disease, demonstrating disturbances in energy, lipid, amino acid, and redox metabolism [6,7,8,9,10,11,12,13,14,15,16]. Metabolic dimensions of ME/CFS may be related to sex; women are disproportionately affected by ME/CFS [1,17]. In analyses of plasma samples, Naviaux et al. (2016) [15] found differences in metabolic pathway disturbances and altered metabolite levels when stratifying ME/CFS cases by sex. Others have also reported sex-specific differences in plasma biomarkers [14,18,19].

Comorbid gastrointestinal (GI) symptoms constitute a potential subtype in ME/CFS [10,12,14,15,18,20,21,22,23]. Among those with ME/CFS, the presence or absence of self-reported irritable bowel syndrome (sr-IBS), in particular, has highlighted differences in the plasma proteome relating to immune dysregulation and altered levels of metabolites within the metabolome [14,18]. In a fecal metagenomics study, Nagy-Szakal et al. (2017) identified eleven bacterial species delineating differences between ME/CFS patients with and without sr-IBS and found relations between bacterial taxa and symptoms relating to fatigue and pain [23].

In this study, we report targeted and untargeted analyses of 888 metabolic analytes comprising of primary metabolites (PM), biogenic amines (BA), complex lipids (CL), and oxylipins (OL) in plasma of ME/CFS cases and controls. We identified altered metabolomic profiles between ME/CFS patients, controls, and subgroups within ME/CFS patients based on sex and sr-IBS.

## 2. Results

### 2.1. Study Population Characteristics

**Figure 1:**
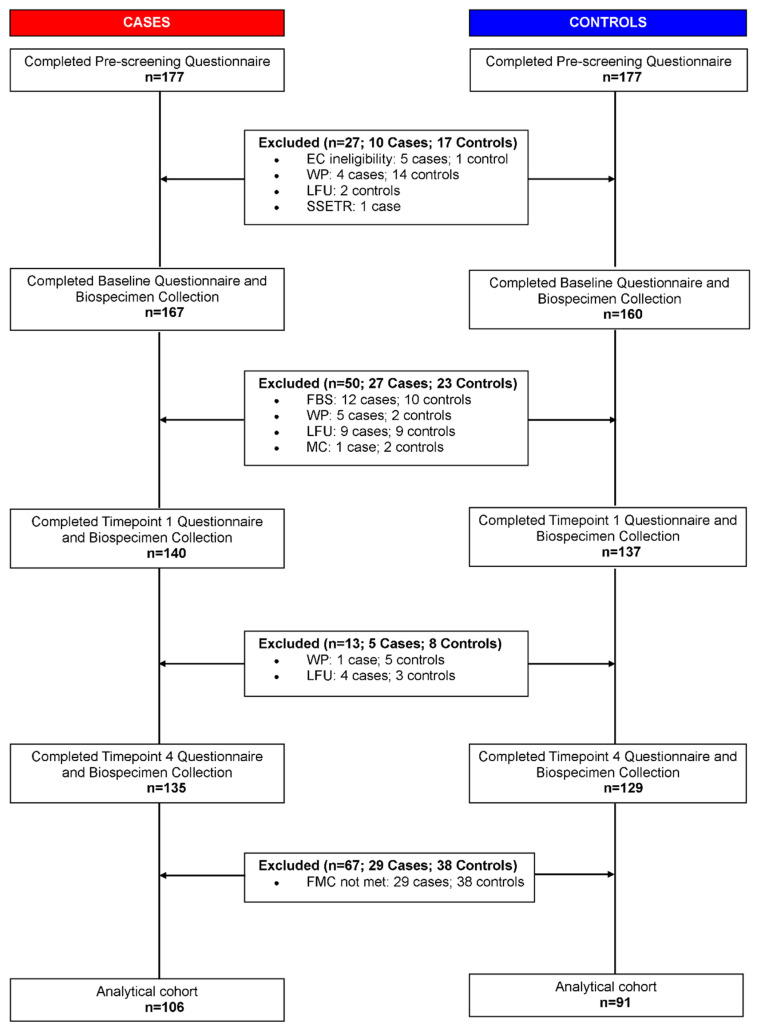
Pipeline for sample selection. EC: Exclusion criteria; WP: Withdrew participation; LFU: Loss to follow up; SSETR: Study site enrollment target reached; FBS: Failed baseline screening; MC: Medical conditions; FMC: Frequency matching criteria.

The study included plasma samples from 106 ME/CFS cases and 91 healthy controls (Figure 1) recruited from five sites across the United States. Demographic and clinical characteristics of the study population are shown in Table 1. ME/CFS cases and controls were similar for all the frequency matching variables except season of collection (*Chi-squared p* = 0.004). We adjusted for all the matching variables (sex, age, race/ethnicity, geographic/clinical site, and season of collection), body mass index (BMI) and sr-IBS in our statistical analyses to account for confounding. All scales in the short form 36 health survey (SF-36) and the multidimensional fatigue inventory (MFI) were significantly different between the two cohorts (*Wilcoxon rank-sum p* < 0.001). The study population is similar to the prescreened cohort that consisted of 177 ME/CFS cases and 177 controls in sex (*Chi-squared p* = 0.60), race (*Chi-squared p* = 0.66) and age (*Wilcoxon rank-sum p* = 0.65).

### 2.2. Metabolomic Dataset

Targeted and untargeted mass spectrometry platforms yielded data for 888 metabolic analytes comprising 100 primary metabolites (PM), 237 biogenic amines (BA), 480 complex lipids (CL), and 71 oxylipins (OL). Supplementary Table S1 shows the sample mean and the standard deviation (SD) of levels of each metabolite within all ME/CFS cases, all controls, female ME/CFS cases, female controls, male ME/CFS cases, male controls, ME/CFS cases without sr-IBS and controls without sr-IBS.

### 2.3. ME/CFS Associated with Altered Metabolomic Profile

In PM, BA, and CL panels, lognormal regression models with log-transformed metabolite levels as dependent variables had the lowest Bayesian information criterion (BIC) values and best fit the data. The estimated coefficients can be interpreted as the differences in the mean values of the log-log transformation of metabolite levels between cases and controls. In OL panel, a mixture of lognormal and log-link Gamma regression models with original metabolite levels as dependent variables best fit the data. For lognormal regression models, the estimated coefficients are interpreted as the mean differences of log transformation of metabolite levels between two groups. For log-link Gamma regression models, the estimated coefficients are interpreted as the log of fold change between two groups. We considered a metabolite to be associated with ME/CFS if it satisfied the following criteria: (1) false discovery rate (FDR) adjusted *p*-value < 0.15; (2) Bayes factor [24] (BF) > 3, and (3) 95% highest density credible interval (HDI) not covering 0. Jeffreys (1961) [25] suggested that the strength of evidence for the alternative hypothesis compared to the null hypothesis is regarded as noteworthy if BFs are above 3.

We did not identify any metabolite as significantly associated with ME/CFS in the PM panel. In the BA panel, levels of acetaminophen were increased in ME/CFS cases compared to controls. In the CL panel, we found decreased levels of plasmalogens, unsaturated phospholipid ethers (PLE), unsaturated phosphatidylcholines (PC), an unsaturated sphingomyelin (SM), and an unsaturated lysophosphatidylcholines (LPC) in ME/CFS cases compared to controls. In the OL panel, decreased levels of Resolvin D1 were observed in ME/CFS cases compared to controls. Table 2 shows the estimated coefficients in the regression models of these metabolites, their associated 95% confidence intervals (CIs), *p*-values, FDR adjusted *p*-values and BFs. Because we used weakly informative priors in Bayesian analysis, the 95% HDIs were similar to the 95% CIs. We report estimations of HDIs in Supplementary Table S2 where estimations for all metabolites are shown.

**Figure 2:**
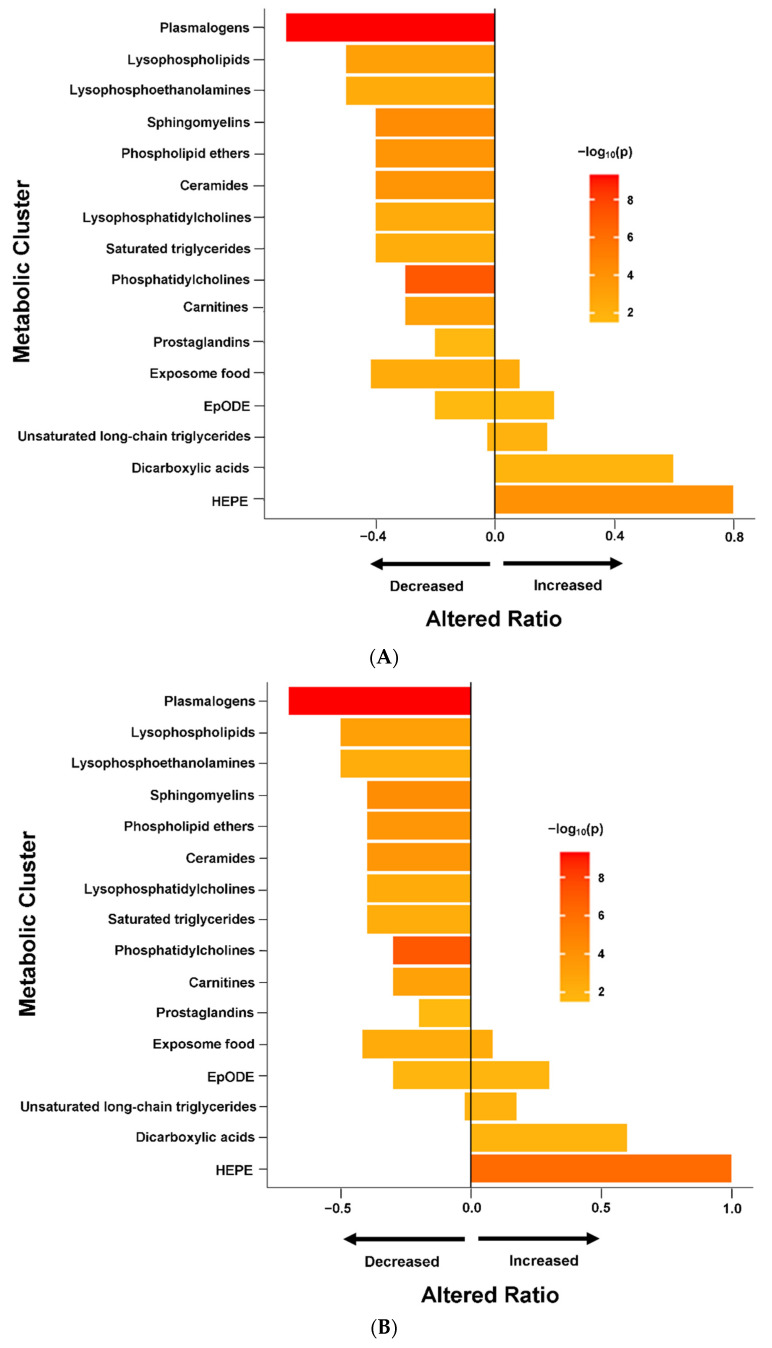

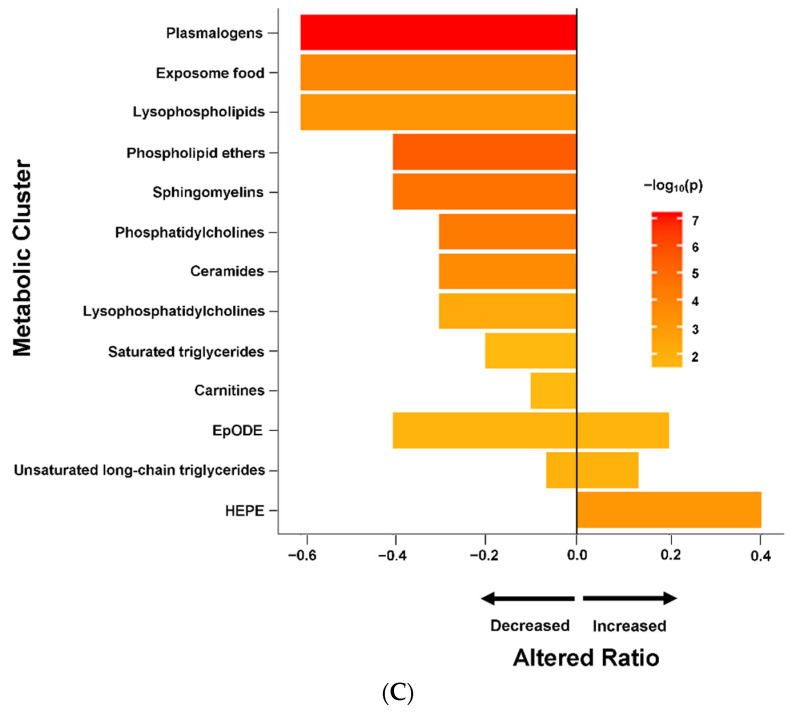
Chemical enrichment analyses using ChemRICH. HEPE: hydroxy eicosapentaenoic acid; ME/CFS: myalgic encephalomyelitis/chronic fatigue syndrome; sr-IBS: self-reported physician diagnosed irritable bowel syndrome. The length of the bar represents altered ratio for each metabolic cluster. A bar restricted to the left of the centered vertical line indicates a metabolic cluster that is lower in ME/CFS patients. A bar restricted to the right of the centered vertical line indicates a metabolic cluster that is higher in ME/CFS patients. A bar that crosses the vertical line indicates a metabolic cluster that is dysregulated in mixed directions. (**A**) All ME/CFS vs. controls. (**B**) Female ME/CFS vs. female controls. (**C**) ME/CFS without sr-IBS vs. controls without sr-IBS.

Set enrichment analysis of the results from the regression models (Figure 2A) revealed that ME/CFS subjects had reduced levels of plasmalogens, sphingomyelins, unsaturated phospholipid ethers, unsaturated ceramides, carnitines, saturated lysophospholipids, unsaturated lysophosphoethanolamines, unsaturated lysophosphatidylcholines, saturated triglycerides and prostaglandins. The majority of unsaturated phosphatidylcholines were also down-regulated in ME/CFS cases. Increased levels of hydroxy-eicosapentaenoic acid (HEPE), dicarboxylic acids, and the majority of unsaturated long chain triglycerides (TG) were found in ME/CFS cases compared to controls. There were mixed directional alterations in the food exposome and epoxy fatty acids (EpODE). Complete data from ChemRICH enrichment analysis are provided in Supplementary Table S3. Data from compound-level enrichment analysis for the significantly altered metabolic clusters are illustrated in Supplementary Table S4. Levels of choline in food exposome were reduced in ME/CFS (estimated coefficient β = −0.009, *p* = 0.004), and only one subject, a control, reported taking choline supplementations in the baseline questionnaire. Levels of succinic acid (β = 0.022, *p* = 0.007) and alpha-ketoglutarate (β = 0.016, *p* = 0.048) in dicarboxylic acids were elevated in ME/CFS.

### 2.4. Altered Metabolomic Profiles in Female and Male ME/CFS Patients

Naviaux et al. (2016) [15] reported that women with ME/CFS, but not men, had disturbed fatty acid and endocannabinoid metabolism. Accordingly, we repeated separately the analyses in female and male cohorts in our study population.

In female subjects, regression and Bayesian analyses (Table 2) revealed that levels of unsaturated PC, plasmalogens, unsaturated phospholipid ethers (PLE) and a single SM in the CL panel were decreased in ME/CFS patients compared to controls. In the BA panel, levels of two drug metabolites, alprazolam and acyclovir, were up-regulated in ME/CFS patients. We did not find the elevated levels of acetaminophen in female subjects (estimated coefficient β = 0.064, FDR adjusted *p* = 0.211, BF = 1.172, 95% HDI = 0.019~0.116) that were observed in the entire ME/CFS (male and female) population. Enrichment analysis in female subjects (Figure 2B) identified dysregulations in the same metabolic clusters as in the overall population. Complete data from enrichment analysis in female subjects are shown in Supplementary Table S5. In contrast, we did not find any metabolites significantly associated with risk of ME/CFS in male subjects. Supplementary Table S6 shows the regression and Bayesian estimations for all metabolites in male and female cohorts.

### 2.5. Altered Metabolomics Profile in ME/CFS Patients without sr-IBS

Due to the limited sample size of subjects with sr-IBS (35 ME/CFS cases and three controls), we only compared levels of metabolites between ME/CFS cases without sr-IBS and controls without sr-IBS. Levels of unsaturated PC, plasmalogens and PLE were decreased in ME/CFS patients in this subgroup (Table 2). In the ChemRICH enrichment analysis, the dysregulations in metabolite clusters found to be dysregulated in the subgroup without sr-IBS (Figure 2C) were all identified in the overall population (Figure 2A). Complete data pertaining to the regression, Bayesian and enrichment analyses are shown in Supplementary Tables S7 and S8.

### 2.6. Machine Learning Analyses

**Figure 3:**
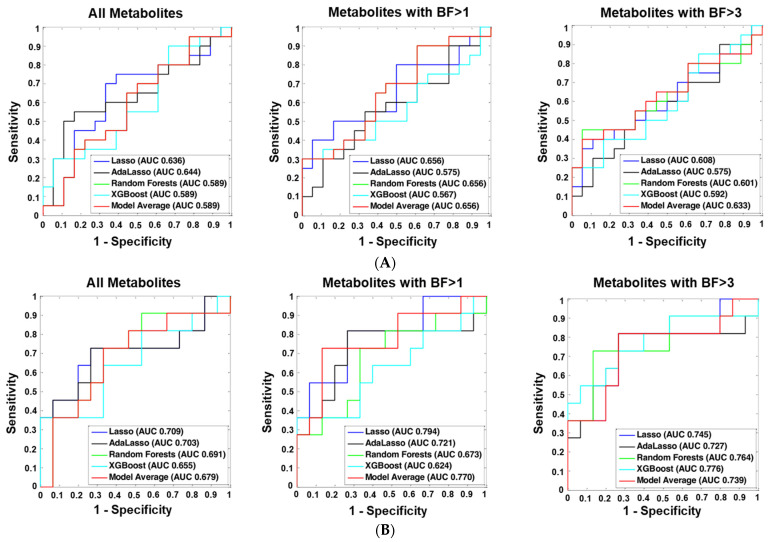

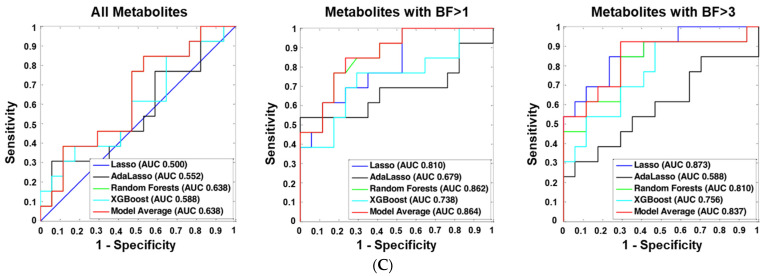
ME/CFS predictive modeling. ME/CFS: myalgic encephalomyelitis/chronic fatigue syndrome; sr-IBS: self-reported physician diagnosed irritable bowel syndrome; BF: BayesFactor; AUC: area under the receiver operating characteristic curve. To differentiate ME/CFS cases from healthy controls, we employed five machine learning algorithms: least absolute shrinkage and selection operator (Lasso), adaptive Lasso (AdaLasso), Random Forests (RF), XGBoost, and Bayesian Model Averaging (Model average). For each algorithm, three sets of predictors were considered: (1) all metabolites, (2) metabolites with BayesFactor > 1, and (3) metabolites with BayesFactor > 3. The predictive models were first trained in the 80% randomly selected training set using 10-fold cross-validation, and the remaining 20% of the study population was used as the independent test set to validate model performance. (**A**) Overall population. (**B**) Women only. (**C**) No GI complaints.

We considered three sets of metabolites as predictors to distinguish ME/CFS cases from controls, including all metabolites, metabolites with BF > 1 and metabolites with BF > 3. Each set of predictors was fitted in five different machine learning classifiers: least absolute shrinkage and selection operator (Lasso) [26], adaptive Lasso (AdaLasso) [27], Random Forests (RF) [28], XGBoost [29], and Bayesian model averaging (Model Average). The classifiers were first trained in the 80% randomly-selected training set and then validated in the remaining 20% test set. Figure 3A–C show the receiver operating characteristic (ROC) curves and the area under the receiver operating characteristic curve (AUC) values differentiating all ME/CFS cases from all controls, female ME/CFS from female controls, and ME/CFS without sr-IBS from controls without sr-IBS, respectively, in the test set. Although classifiers did not differentiate all ME/CFS from all controls, Lasso with BF > 1 metabolites as predictors distinguished female ME/CFS patients from female controls with an AUC value of 0.794 (95% CI: 0.612–0.976), and Lasso with BF > 3 metabolites distinguished ME/CFS without sr-IBS from controls without sr-IBS with an AUC value of 0.873 (95% CI: 0.747–0.999). The AUC values and their associated 95% CIs of all the classifiers are shown in Supplementary Table S9, and the true/false positive/negative rates of the best performing classifiers are shown in Supplementary Table S10.

### 2.7. Correlations between Metabolites and ME/CFS Symptom Severity Scores

**Figure 4:**
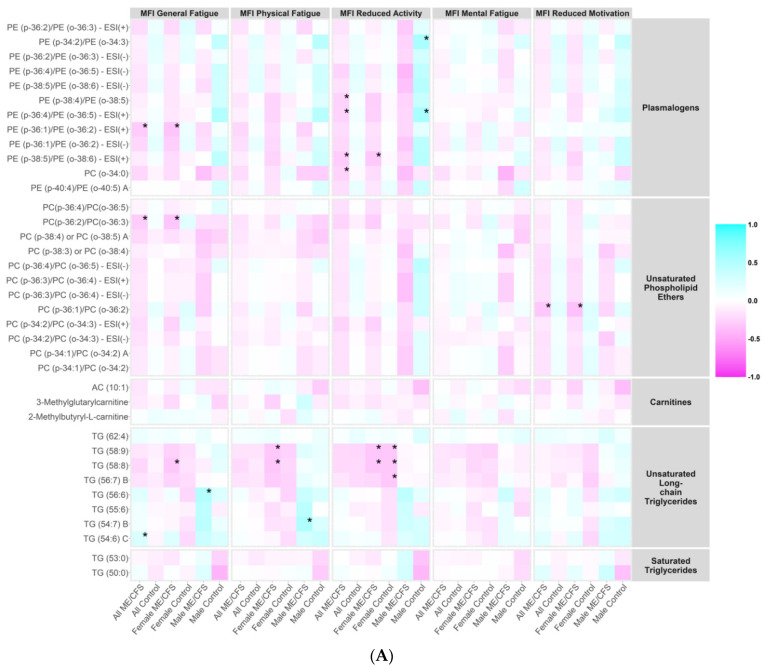

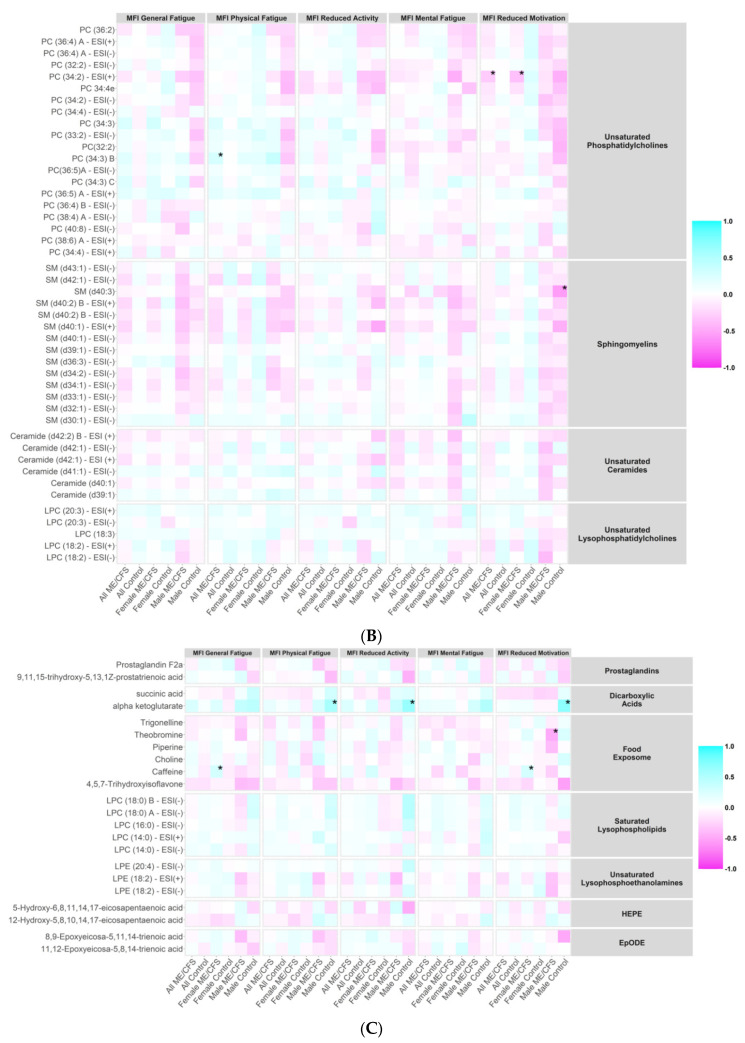
Correlation heatmap. MFI, Multidimensional Fatigue Inventory scored on 0–100 scale with 0 = no fatigue and 100 = maximal fatigue. ME/CFS: myalgic encephalomyelitis/chronic fatigue syndrome. * *p* < 0.01. Heatmap showing the correlation coefficients between the plasma levels of metabolites in the metabolic clusters that were significantly altered in ME/CFS (bold in Supplementary Table S4) and MFI scales using Spearman’s correlation tests in all ME/CFS, all controls, female ME/CFS, female controls, male ME/CFS, and male controls. (**A**) Correlation heatmap, part 1. (**B**) Correlation heatmap, part 2. (**C**) Correlation heatmap, part 3.

We investigated whether the plasma levels of metabolites in the metabolic clusters that were significantly altered in ME/CFS (bold in Supplementary Table S4) correlated with the MFI scales using Spearman’s correlation tests. Heatmaps showing the correlation coefficients in all ME/CFS, all controls, female ME/CFS, female controls, male ME/CFS, and male controls are presented in Figure 4A–C.

Notably, within male ME/CFS patients, levels of TG (56:6) in unsaturated long-chain TG were positively correlated with the MFI general fatigue scales (ρ = 0.501, *p* = 0.005), and levels of TG (54:7) B were positively correlated with the MFI physical fatigue scales (ρ = 0.519, *p* = 0.003). Within male controls, levels of alpha-ketoglutarate (α-KG) in dicarboxylic acids were positively correlated with the MFI physical fatigue (ρ = 0.558, *p* = 0.007), reduced activity (ρ = 0.633, *p* = 0.002) and reduced motivation (ρ = 0.696, *p* < 0.001) scales. More severe MFI reduced activity symptoms were associated with higher levels of two plasmalogens (PE (p-34:2)/PE (o-34:3): ρ = 0.549, *p* = 0.010; PE (p-36:4)/PE (o-36:5)—ESI (+): ρ = 0.551, *p* = 0.010). Levels of SM (d40:3) in sphingomyelins were negatively correlated with the MFI reduced activity scales (ρ = −0.557 *p* = 0.009).

## 3. Discussion

Since the first reports of large-scale metabolomic studies in people with ME/CFS were published in 2016 by Naviaux [15], several research teams, including Yamano [16], Fluge [9], Hoel [30], and our own [14], have reported metabolomic analyses of plasma. The common threads in the results from all these studies are decreased levels of phospholipids and metabolic dysregulation, suggesting abnormalities in lipid remodeling activity that impair oxidative metabolism. In the present study, we also observed decreased levels of phospholipids, especially plasmalogens and phospholipid ethers. Furthermore, consistent with previous literature [6,16,30], our results suggest dysregulation of peroxisomal metabolism and the tricarboxylic acid (TCA) cycle.

### 3.1. Lipid Metabolism Abnormalities

Regression, Bayesian, and enrichment analyses revealed significant reduction in the levels of plasmalogens in the ME/CFS group compared to the control group. Plasmalogens are abundant phospholipid ethers that protect phospholipids and lipoprotein particles from oxidative stress and associated damage [31,32]. They are also responsible for maintaining the integrity of membrane structures [31]. Plasmalogen biosynthesis commences in the peroxisomes and is completed in the endoplasmic reticulum [33]. Maintenance of peroxisomal structure with functional enzymes is imperative for both plasmalogen biosynthesis and β-oxidation of very long-chain fatty acids [31]. Peroxisomal β-oxidation of very long-chain fatty acids leads to their breakdown into short-chain products that serve as substrates for mitochondrial β-oxidation [34]. We posit that this crosstalk between mitochondria and peroxisomes plays an important role in maintaining energy homeostasis, and that dysregulation contributes to the fatigue and cognitive dysfunction that are hallmarks of ME/CFS [35,36].

ME/CFS subjects had a significant reduction in levels of carnitines (Figure 2A–C). Carnitines regulate the cellular to mitochondrial ratio of free CoA to acyl-CoA, remove the unwanted acyl groups, and play a key role in the transport of long-chain fatty acids from cytoplasm to the mitochondrial matrix for oxidation [37]. Depletion of carnitines can threaten the integrity of cell and mitochondrial membranes, increase oxidative stress, and reduce the ability to counter inflammation [38]. We also observed increased levels of long-chain triglycerides in ME/CFS. Depletion of carnitines leads to the accumulation of long-chain triglycerides that become targets for lipid peroxidation by mitochondria [39]. The accumulation of toxic lipid peroxidation products can also lead to mitochondrial membrane damage [19]. Where carnitine is depleted and there is mitochondrial overload for fatty acid oxidation, peroxisomal β-oxidation has been reported to be a compensatory process that can produce carnitine as an intermediate product [40].

Peroxisomes regulate fatty acid metabolism through metabolic cross-talk with mitochondria [41]. Missailidis et al. (2021) [35] have reported potential dysregulation in mitochondrial β-oxidation in conjunction with dysregulation in peroxisomal processes in lymphoblasts of ME/CFS patients. Our findings of depleted levels of plasmalogens, unsaturated phospholipid ethers and carnitines are consistent with peroxisomal dysfunction. Peroxisomes also regulate the scavenging of reactive oxygen species (ROS). Redox imbalance is frequently seen in people with ME/CFS [42]. The peroxisomal dysfunction we observed could contribute to and/or reflect this redox imbalance. Finally, peroxisomes are critical in maintaining membrane integrity.

We found depleted levels of phosphatidylcholines (PCs) in ME/CFS subjects. PCs are abundant phospholipids in the mitochondrial membranes [43,44]. Most PCs are synthesized via the CDP-choline pathway [45] and may undergo substantial lipid remodeling via lipases and acyltransferases. PCs are essential to the formation of intermediate structures in membrane fusion and fission events, for stabilizing mem brane proteins into their optimal conformations, and for actin-filament disassembly in the end stage of cytokinesis [46,47,48]. One critical functional implication of reduced levels of PCs is impaired oxidative phosphorylation. PC depletion specifically affects the function and stability of the protein translocases of mitochondria, including the inner membrane translocase TIM23 complex [49] and the outer membrane sorting and assembly machinery (SAM) complex [49,50]. The destabilization of TIM23 and SAM complexes leads to reduction in mitochondrial membrane potential and impair protein transport and respiratory chain activities [49].

ME/CFS patients were also found to have decreased levels of ceramides, sphingomyelins, lysophosphatidylcholines, phospholipid ethers, prostaglandin D2 (PGD2) and prostaglandin F2α (PGF2α). Depleted levels of lysophosphatidylcholines and phospholipid ethers, as well as depleted levels of PCs, can impede mitochondrial respiration [46]. Reduced synthesis of PGF2α and PGD2 in phospholipase A2γ-deficient mice induces mitochondrial dysfunction as well as oxidative stress, which can contribute to further mitochondrial damage [51]. PCs, ceramides, sphingomyelins, and phospholipid ethers are important components of the lipid bilayer, and reduction in their levels dysregulate signal transduction across membranes. This alteration in the levels or conformation of membrane components can adversely affect the function of proteins, such as G protein coupled receptors (GPCRs), embedded in the membranes [52]. Phospholipids can act as direct allosteric modulators of GPCR activity through the lipid head group that affects ligand binding (agonist and antagonist) and receptor activation [52]. In addition, PCs are precursors to many biologically active molecules that can act as second messengers. Prominent among them are diacylglycerol (DAG), fatty acids, phosphatidic acid, lysophosphatidic acid, N-arachidonylethanolamine, N-palmitoylethanolamine, N-steroylethanolamine and arachidonic acid [53,54,55]. Ceramides are not only structural components of membranes but can also act as second messengers in modulating a range of cellular signaling pathways [56].

Depletions in levels of choline approached, but did not meet, the association criteria (adjusted *p* = 0.139, BF = 2.75, 95% HDI=−0.015~−0.003). Choline is an important nutrient; 95% of it is utilized in the synthesis of PCs via the CDP-choline pathway [45]. The remaining 5% exists as either free choline or is used in the synthesis of phosphocholine, glycerophosphocholine, CDP-choline, acetylcholine, and other choline-containing phospholipids like sphingomyelin, plasmalogens and lysophosphatidylcholine. Each of these compounds contributes to maintenance of the structure and signaling functionality of the plasma membrane [45,53]. IgG autoantibodies that specifically target GPCRs have been reported, even in healthy individuals, but are more commonly found in ME/CFS [57,58], particularly to autonomic nervous system targets including the M3 Acetylcholine receptor (M3AChR) and β2 Adrenergic receptor (β2AdR). Agonists for each of these receptors have choline precursors, acetylcholine (AC) and epinephrine (adrenaline), respectively. Choline also plays a role in the production of epinephrine by donating the methyl group. Thus, choline deficiency could potentially lead to the autonomic dysfunction that is found in many people with ME/CFS, with reduced tissue blood flow and oxygen supply, leading to hypoxia, ischemia and fatigue [59]. Depletion in the levels of choline, PCs, ceramides, sphingomyelins and lysophosphatidylcholines, suggest dysregulation of the CDP-choline pathway.

### 3.2. TCA Cycle and Other Abnormalities

Through enrichment analysis, we found significant elevations in the levels of dicarboxylic acids in ME/CFS subjects. The two TCA cycle intermediates, alpha-ketoglutarate (α-KG) and succinate, representing the dicarboxylic acids cluster, were elevated in ME/CFS. The TCA cycle is a conserved pathway in aerobic organisms through which the acetyl-CoA from carbohydrates, fats and proteins is converted into ATP [60]. Increased levels of α-KG have been reported previously in ME/CFS patients [12], although we are not aware of previous reports of elevated levels of succinate. Abnormal levels of TCA cycle intermediates suggest inefficiencies in ATP production that may contribute to the fatigue and post-exertional malaise reported in ME/CFS. Increases in α-KG levels have been reported to induce severe metabolic impairment of pyruvate oxidation in the tricarboxylic acid cycle, leading to cell death [60]. Succinate accumulation has been reported to induce HIF-1α stabilization as well as the transcriptional activation of the pro-inflammatory cytokine IL-1β [60]. Elevated succinate levels contribute to increased oxidative stress and neuronal degeneration in rat models [61]. Oxidative stress, in turn, augments nitrosative stress [62]. Nitrosative stress, which has been documented in people with ME/CFS [42,63], can lead to the increased production of peroxynitrite and downregulate the function of both α-KG dehydrogenase and succinate dehydrogenase [62,63,64]. Infection is a common cause of nitrosative stress. Many ME/CFS patients report symptoms consistent with systemic infection prior to the onset of the illness.

Our analyses also revealed reductions in levels of Resolvin D1 in ME/CFS. Resolvin D1 is a derivative of docosahexanoic acid (DHA) and contributes to resolution of inflammation by targeting dead cells for clearance by macrophages [65]. Decreased levels of Resolvin D1 in ME/CFS may be consistent with the possibilities of inflammatory damage associated with the disease [66,67].

### 3.3. Metabolomic Findings as Biomarkers of Disease and of Disease Severity

To identify biomarkers for ME/CFS, we explored three sets of predictors and five different machine learning models. None of the classifiers differentiated all ME/CFS subjects from controls; however, the predictive performance of our subgroup analyses was better in female and no sr-IBS sub-cohorts than in the overall population. This is consistent with earlier findings in our ME/CFS studies and likely reflects heterogeneity in both phenotype and pathogenesis [14,18,23]. We further tested their predictive capacities with an independent cohort whose metabolomics profiling we previously explored [14]. The metabolomics assay of the validation set was matched with our current assay, resulting in 630 metabolites in common. The metabolites that overlapped with the three sets of predictors (all, BF > 1, BF > 3) were fitted into the same machine learning models, and the predictive performance was evaluated using 10-fold cross-validation in the validation dataset. The AUC values for distinguishing all ME/CFS patients from all controls ranged between 0.514 and 0.738. For differentiating female ME/CFS subjects from female controls, the AUC values were between 0.616 and 0.784; for differentiating ME/CFS patients without sr-IBS from controls without sr-IBS, the AUC values ranged between 0.614 and 0.828. The predictive performance was similar in the validation set, as observed in the current study (Supplementary Table S9), and in models with larger BFs, as predictors also performed better than those fitted with all metabolites.

From Figure 4A, we observed generally higher correlation coefficients in all peroxisome-related metabolites (plasmalogen, phospholipid ethers, carnitines, and long-chain triglycerides) with the energy MFI scales (General Fatigue, Physical Fatigue, and Reduced Activity) than with the mental MFI scales (Mental Fatigue and Reduced Motivation). Plasmalogens and phospholipid ethers, in particular, are directly linked to peroxisomal disorders, and were generally negatively correlated with the energy MFI scales in ME/CFS and ME/CFS subgroups. This is consistent with the findings in the statistical analyses that levels of these compounds were depleted in ME/CFS compared to controls, and the stronger correlations in the energy MFI scales provided further support to the notion that dysregulated interactions between mitochondria and peroxisomes contribute to fatigue in ME/CFS [35]. The correlation coefficients in the male cohort were generally larger than those in the female cohort; however, the correlation coefficient estimates are prone to bias especially when the sample size is small (31 male cases and 22 male controls vs. 75 female cases and 69 female controls).

### 3.4. Strengths and Limitations

The strength of this study lies in the quality of patient characterization, robust metabolomic analysis involving a comprehensive set of compounds, and complete and cautious statistical approaches. The three association criteria that combine inferences from frequentist and Bayesian analyses enhance the robustness of our findings without compromise on the sensitivity. Although our current study has a larger sample size than many previously published metabolomics studies in ME/CFS, it is imperative that the validity of novel findings reported here be independently tested in other cohorts. Our subgroup analyses focusing on female subjects and subjects without sr-IBS did not reveal dysregulated metabolic clusters different from those in the overall study population. These analyses were limited by small sample sizes in the subgroups of male subjects and subjects with sr-IBS. The analyses correlating metabolite levels with ME/CFS symptom scores were limited by the subjective report of MFI instrument.

## 4. Materials and Methods

### 4.1. Study Population

Our starting population comprised 177 ME/CFS cases and 177 controls in ME/CFS clinics in Incline Village, NV; Miami FL; New York, NY; Salt Lake City, UT; and Palo Alto, CA. All ME/CFS cases met the 1994 CDC Fukuda [68] and Canadian consensus criteria for ME/CFS [69], and were rendered with ME/CFS diagnosis from a clinician. All ME/CFS cases completed standardized screening and assessment instruments including medical history and symptom rating scales as well as a physical examination. Controls were matched to cases on age, sex, race/ethnicity, geographic/clinical site, and date of sampling (±30 days). Based on screening criteria, we excluded five ME/CFS cases that met any exclusion criteria from the 1994 CDC Fukuda and/or Canadian consensus criteria for ME/CFS, such as having chronic infections, rheumatic and chronic inflammatory diseases, neurological disorders, psychiatric conditions, or were taking any immunomodulatory medication. Controls underwent the same screening process as ME/CFS subjects and were excluded if they reported ME/CFS or other conditions deemed by the recruiting physician to be inconsistent with a healthy control population. Controls were also excluded if they had a history of substance abuse, psychiatric illness, antibiotics in the prior three months, immunomodulatory medications in the prior year, and clinically significant findings on physical exam or screening laboratory tests. One control was excluded after prescreening based on these criteria. Additionally, 21 participants were excluded prior to baseline due to withdrawal from the study (*n* = 18), loss to follow-up (*n* = 2), and enrollment capacity (*n* = 1). The baseline questionnaire was completed by with 327 participants. During the study, an additional 63 participants were excluded for study protocol deviations (*n* = 25), loss to follow-up (*n* = 25), and withdrawal from the study (*n* = 13), resulting in a total of 264 participants.

For the analysis reported here, a sub-cohort was established based on complete survey and biospecimen data (blood, saliva, and stool) at the first and last time points of the study and key demographic characteristics were frequency-matched to ensure that the nested cohort was similar to the full cohort. This sub-cohort consisted of 106 ME/CFS cases and 91 controls; the derivation of the sub-cohort is summarized in Figure 1. All participants provided informed written consent in accordance with protocols approved by the Institutional Review Board at Columbia University Irving Medical Center.

### 4.2. Plasma Collection

All participants fasted from midnight prior to the sample collection. Blood samples were collected into BD VacutainerTM Cell Preparation Tubes (CPT) with ethylenediaminetetraacetic acid (EDTA) anticoagulant between January 2016 and June 2016, and centrifuged to pellet red blood cells. The plasma was shipped to Columbia University at 4 °C. After aliquoting, samples were stored at −80 °C until thawed for metabolomics analyses. All the samples were analyzed within two years of collection.

### 4.3. Clinical Assessment

Clinical symptoms and baseline health status were assessed on the day of physical examination and biological sample collection from both case and control subjects using the following instruments: the Short Form 36 Health Survey (SF-36), the Multidimensional Fatigue Inventory (MFI), DePaul Symptom Questionnaire (DSQ) [70], and Pittsburgh Sleep Quality Index (PSQI) [71]. The SF-36 includes the following subject-reported evaluations about current health status: physical and social functioning, physical and emotional limitations, vitality, pain, and general and mental health [72]. The MFI comprises of a 20-item self-reported questionnaire focused on general, physical and mental fatigue, reduced activity, and reduced motivation [73]. Cognitive function was tested based on the self-reported DSQ questionnaire data, and was scored using a standard cognitive disturbance definition as well as a modified definition based on a subset of questionnaire variables. Sleeping disturbances linked to ME/CFS were tested and scored based on DSQ and PSQI questionnaire items. Each instrument was transformed into a 0–100 scale to facilitate combination and comparison. The questionnaire reflecting these instruments is included in the Supplementary Materials.

A diagnosis of sr-IBS was based on answers in the medical history form. Subjects were asked if they had received a previous IBS diagnosis by a physician and the date of that diagnosis. Of the 106 subjects with ME/CFS, 35 (33.0%) had sr-IBS. Of the 91 control subjects, 3 (3.3%) had sr-IBS.

### 4.4. Metabolomics Analysis

Samples were stored at −80 °C before analysis. Untargeted metabolomics data were acquired using three chromatography/mass spectrometry-based assays (MS). (1) Primary metabolites such as mono- and disaccharides, hydroxyl- and amino acids were measured by gas chromatography/time-of-flight mass spectrometry (GC-TOF MS) [74] including data alignment and compound annotation using the BinBase database algorithm [75]. (2) Biogenic amines including microbial compounds such as trimethylamine N-oxide (TMAO), methylated and acetylated amino acids and short di- and tripeptides were measured by hydrophilic interaction liquid chromatography/quadrupole time-of-flight mass spectrometry (HILIC-QTOF MS). (3) Complex lipids including phosphoglycerolipids, triacylglycerides, sphingolipids, and free fatty acids were analyzed by liquid chromatography (LC)/quadrupole time-of-flight mass spectrometry (CSH-QTOF MS) [76]. Targeted bioactive oxylipin assay included thromboxanes, prostaglandins, and hydroxy-, keto- and epoxy-lipins. All LC-MS/MS data included diverse sets of internal standards. LC-MS data were processed by MS-DIAL vs. 4.0 software [77], and the compounds were annotated based on accurate mass, retention time and MS/MS fragment matching using LipidBlast [78] and Massbank of North America libraries [79]. MS-FLO was used to remove erroneous peaks and reduce the false discovery rate in LC datasets [80]. A total of 821 known metabolites were annotated. Some complex lipids were annotated in both positive (ESI+) and negative (ESI−) ion modes, resulting in a total of 888 metabolic analytes that were included in our analysis. Data were normalized by SERRF [81]. Residual technical errors were assessed by coefficients of variation (CV) for known metabolites.

### 4.5. Statistical Analyses

For each metabolic analyte, zero values reflecting a measurement below the detection limit were replaced with 50% of its smallest available value. In each of the four metabolomics panels, outliers were identified through principal component analysis (PCA). In PM, six outliers (four cases and two controls) were identified and removed; in CL, there were five outliers (three cases and two controls); in OL, there was one outlier (one case); in BA, four outliers (three cases and one control) were eliminated.

To compare the levels of each metabolite between ME/CFS cases and controls, we employed a variety of regression models with the metabolite level as the dependent variable and the binary case/control status as the independent variable, adjusting for all the matching variables (age, sex, race/ethnicity, geographic/clinical site, and season of sampling), BMI and sr-IBS. We considered two options for the dependent variable: (1) original metabolite levels, and (2) natural log-transformed metabolite levels. Before log-transformation, if necessary, all data points in metabolic analytes were multiplied by a minimal factor to keep the feature on a positive domain. Four regression models were considered: Gaussian regression with identity link, Gaussian regression with log link, lognormal regression and Gamma regression with log link. The BIC was used to select the best fitting transformation/regression combination. We then calculated the estimated coefficient for the case/control status, together with its 95% confidence interval (95% CI) and *p*-value. Multiple comparisons over all metabolites were corrected using the Benjamini-Hochberg procedure [82] controlling the FDR at the 0.15 level. Additionally, chemical enrichment analyses were performed using ChemRICH [83] to determine chemical classes that were significantly altered between groups. ChemRICH does not rely upon background databases for statistical calculations and provides enrichment analysis based upon chemical structure, as opposed to defined pathways that can be inherently flawed [83].

For each metabolite, we also conducted Bayesian analysis with the best fitting transformation/regression combination using R packages “rstanarm” [84] and “bayestestR” [85]. Default (weakly informative) prior distributions from rstanarm were applied adjusting the scales of the priors internally. The default priors do not strongly affect the posterior distribution but help stabilize computation, while still allowing for extreme effect sizes if warranted by the data [86,87]. We then calculated the Bayes factors (BFs) and 95% highest density credible intervals (HDIs). The BF of a single parameter indicates the degree by which the mass of the posterior distribution has shifted further away from or closer to the null value (zero), relative to the prior distribution [24]. Hence, the BF measures the strength of evidence in favor of the alternative hypothesis (β ≠ 0) over the null hypothesis (β = 0). The 95% credible interval in the Bayesian framework is the range within which the effect has 95% probability of falling, given the observed data; it has a different interpretation from the 95% confidence interval in the frequentist framework which instead signifies that with a large number of repeated samples, 95% of such calculated confidence intervals would include the true value of the parameter. We considered a metabolite to be associated with ME/CFS if it satisfied the following criteria: (1) FDR adjusted *p*-value < 0.15, (2) BF > 3, and (3) 95% HDIs not covering 0.

Naviaux et al. (2016) [15] showed that potential diagnostic metabolites for ME/CFS in targeted metabolomics are different between male and female subjects. Accordingly, we conducted sex-stratified analyses in addition to analyses with the whole cohort. In our previous work with a different cohort, sr-IBS comorbidity was identified as the strongest driving factor in the separation of topological networks based on fecal microbiome and plasma metabolic pathways [14,23]. We subsequently found different patterns in the relationships between plasma proteomic profiling and ME/CFS when comparing ME/CFS with or without sr-IBS to healthy controls [18]. Given this precedent, we tested the hypothesis that sr-IBS subgroups in ME/CFS patients have altered metabolic profiles in a stratified analysis. As there were only three control subjects with sr-IBS, we focused on the comparison of ME/CFS subjects without sr-IBS versus controls without sr-IBS.

To explore the utility of the metabolomics assay as a biomarker tool for ME/CFS, we employed four machine learning algorithms: Lasso [26], AdaLasso [27], RF [28] and XGBoost [29]. AdaLasso is different from Lasso in that AdaLasso has the oracle property that leads to consistent variable selection whereas Lasso is only consistent for variable selection under certain conditions on the shrinkage parameters and correlations [88]. For each of the algorithm, three sets of predictors were considered: (1) all metabolites, (2) metabolites with BF > 1, and (3) metabolites with BF > 3. The predictive models were first trained in the 80% randomly-selected training set using 10-fold cross-validation; the remaining 20% of the study population was used as the independent test set to validate model performance. We also applied the Bayesian model averaging (Model Average) method [89] that combines the predictions of multiple models using weighted averages in which the weights are Bayesian posterior probabilities that the given model is the true model, conditional on the training data. We assigned equal prior probabilities (1/4) to each of the four models; therefore, the posterior probabilities were proportional to the model likelihood in the 10-fold cross-validation using the training data. The predictive performance of the five models (Lasso, AdaLasso, RF, XGBoost and Model Average) using the three sets of predictors in the test set was evaluated using AUC values and ROC curves.

Data analyses were performed using MATLAB Statistics Toolbox R2013a (MathWorks, Inc., Natick, MA, USA) and R version 3.6.3 (RStudio, Inc., Boston, MA, USA). All *p*-values were 2-tailed.

## 5. Conclusions

Our findings indicate a series of interconnected metabolic alterations in people with ME/CFS that may contribute to the pathogenesis of ME/CFS: (i) reduced levels of plasmalogens, unsaturated phospholipid ethers, and carnitines suggest peroxisomal dysfunction; (ii) reductions in levels of PCs indicate dysregulation of CDP-choline pathway, and (iii) elevations in the levels of dicarboxylic acids, particularly the TCA cycle intermediates alpha-ketoglutarate and succinate, consistent with an impairment in the TCA cycle that may contribute to physical and cognitive fatigue.

## Figures and Tables

**Table 1:** Subject characteristics.

Subject Characteristics		ME/CFS (*n* = 106)	Controls (*n* = 91)	*p*-Value *
Sex	Female	75	69	0.42
	Male	31	22	
Age	Mean ± SD	47.8 ± 13.7	47.0 ± 14.1	0.78
	Median (Range)	51.0 (21.6–70.0)	50.6 (21.2–68.2)	
Race and Ethnicity	White and not Hispanic	93	85	0.40
	Hispanic	6	3	
	Not White and not Hispanic	7	3	
Site of Collection	Incline Village, NV	23	17	0.30
	Miami, FL	15	5	
	New York, NY	17	19	
	Salt Lake City, UT	32	32	
	Palo Alto, CA	19	19	
Season of Collection	Winter	33	14	0.004
	Spring	72	70	
	Summer	1	7	
sr-IBS Comorbidity ^†^	Yes	35	3	<0.001
	No	71	88	
BMI	Mean ± SD	26.1 ± 5.2	25.2 ± 4.7	0.31
	Median (Range)	25.0 (18.1–41.2)	25.1 (16.9–38.7)	
Duration of ME/CFS ^‡^	Mean ± SD	15.0 ± 9.8	n/a	n/a
	Median (Range)	14.4 (1.2–44.2)	n/a	
	≥3 years	92	n/a	
	<3 years	8	n/a	
SF-36 Scales ^§^ Median (IQR)	Emotional Limitations	83.3 (0.0–100.0)	100.0 (100.0–100.0)	<0.001
	Emotional Well-being	72.0 (56.0–84.0)	88.0 (80.0–92.0)	<0.001
	General Health	20.0 (15.0–30.0)	90.0 (75.0–95.0)	<0.001
	Pain	45.0 (22.5–66.25)	90.0 (90.0–100.0)	<0.001
	Physical Functioning	35.0 (20.0–55.0)	100.0 (95.0–100.0)	<0.001
	Physical Limitations	0.0 (0.0–0.0)	100.0 (100.0–100.0)	<0.001
	Social Functioning	22.5 (10.0–45.0)	100.0 (90.0–100.0)	<0.001
	Vitality	5.0 (0.0–20.0)	80.0 (70.0–85.0)	<0.001
MFI Scales ^||^ Median (IQR)	General Fatigue	91.7 (71.9–100.0)	16.7 (4.2–33.3)	<0.001
	Mental Fatigue	62.5 (45.8–75.0)	12.5 (0.0–35.4)	<0.001
	Physical Fatigue	87.5 (66.7–100.0)	12.5 (4.2–20.8)	<0.001
	Reduced Activity	79.2 (58.3–95.8)	4.2 (2.1–29.2)	<0.001
	Reduced Motivation	45.8 (29.2–66.7)	12.5 (4.2–25.0)	<0.001

SD: standard deviation; IQR: interquartile range; ME/CFS: myalgic/encephalomyelitis/chronic fatigue syndrome. * For categorial variable, *p*-values were derived from Chi-squared tests; for continuous variables, *p*-values were derived from Wilcoxon rank-sum tests. ^†^ Prior physician diagnosed irritable bowel syndrome, self-reported on the questionnaire. ^‡^ Only 90 responses were received for this item. ^§^ 36-Item Short Form Health Survey; scored on a 0–100 scale with 0 = poor health status and 100 = excellent health status. ^||^ Multidimensional Fatigue Inventory; scored on 0–100 scale with 0 = no fatigue and 100 = maximal fatigue.

**Table 2:** Metabolites significantly associated with ME/CFS or ME/CFS subgroups.

Metabolite	Enrichment Cluster	Regression Model	ME/CFS vs. Control				
			Estimated Coefficient	95% CI	*p*-Value	FDR	Bayes Factor
Biogenic Amines (BA)							
Acetominophen	drugs	Lognormal	0.068	(0.028, 0.108)	0.001	0.103	3.035
Complex Lipids (CL)							
PE (p-36:2)/PE (o-36:3)—ESI(+)	plasmalogens	Lognormal	−0.028	(−0.043, −0.013)	0.000	0.074	20.935
PE (p-34:2)/PE (o-34:3)	plasmalogens	Lognormal	−0.037	(−0.060, −0.014)	0.002	0.126	5.662
LPC (18:2)—ESI(−)	unsaturated LPC	Lognormal	−0.019	(−0.032, −0.007)	0.003	0.139	4.102
PC (36:2)	unsaturated PC	Lognormal	−0.007	(−0.011, −0.003)	0.000	0.074	11.241
PC (36:4) A—ESI(+)	unsaturated PC	Lognormal	−0.018	(−0.028, −0.008)	0.000	0.074	8.134
PC (36:4) A—ESI(−)	unsaturated PC	Lognormal	−0.019	(−0.031, −0.008)	0.001	0.103	4.032
PC (32:2)—ESI(−)	unsaturated PC	Lognormal	−0.027	(−0.043, −0.010)	0.002	0.135	7.389
PC 34:4e	unsaturated PC	Lognormal	−0.022	(−0.036, −0.008)	0.003	0.139	4.327
PC (p-34:2)/PC (o-34:3)—ESI(+)	unsaturated PLE	Lognormal	−0.018	(−0.027, −0.009)	0.000	0.062	44.620
PC (p-34:1)/PC (o-34:2)	unsaturated PLE	Lognormal	−0.021	(−0.032, −0.010)	0.000	0.062	178.678
PC (p-36:1)/PC (o-36:2)	unsaturated PLE	Lognormal	−0.055	(−0.086, −0.024)	0.001	0.074	11.555
PC (p-34:2)/PC (o-34:3)—ESI(−)	unsaturated PLE	Lognormal	−0.020	(−0.032, −0.009)	0.001	0.074	12.281
PC (p-36:4)/PC (o-36:5)—ESI(−)	unsaturated PLE	Lognormal	−0.021	(−0.034, −0.009)	0.001	0.103	7.046
PC (p-34:1)/PC (o-34:2) A	unsaturated PLE	Lognormal	−0.027	(−0.044, −0.011)	0.002	0.125	5.655
Oxylipins (OL)							
Resolvin D1	OH-FA_22_6_1	Gamma	−0.528	(−0.846, −0.210)	0.002	0.134	6.635
**Metabolite**	**Enrichment** **Cluster**	**Regression Model**	**Female ME/CFS vs. Female Control**				
			**Estimated Coefficient**	**95% CI**	***p*** **-Value**	**FDR**	**Bayes Factor**
Biogenic Amines (BA)							
Alprazolam	drugs	Lognormal	0.081	(0.030, 0.132)	0.002	0.121	3.486
Acyclovir	drugs	Lognormal	0.152	(0.057, 0.247)	0.002	0.121	3.179
Complex Lipids (CL)							
PE (p-36:2)/PE (o-36:3)—ESI(+)	plasmalogens	Lognormal	−0.033	(−0.049, −0.017)	0.000	0.048	24.602
PE (p-34:2)/PE (o-34:3)	plasmalogens	Lognormal	−0.042	(−0.066, −0.018)	0.001	0.064	6.155
SM (d40:3)	sphingomyelins	Lognormal	−0.035	(−0.055, −0.014)	0.001	0.064	6.392
PC (36:2)	unsaturated PC	Lognormal	−0.009	(−0.013, −0.004)	0.000	0.054	14.972
PC (36:4) A—ESI(+)	unsaturated PC	Lognormal	−0.022	(−0.033, −0.010)	0.000	0.054	8.061
PC (36:4) A—ESI(−)	unsaturated PC	Lognormal	−0.025	(−0.038, −0.011)	0.000	0.054	11.432
PC 34:4e	unsaturated PC	Lognormal	−0.029	(−0.045, −0.014)	0.000	0.054	13.135
PC (34:2)—ESI(+)	unsaturated PC	Lognormal	−0.006	(−0.010, −0.003)	0.000	0.054	6.913
PC (p-34:2)/PC (o-34:3)—ESI(+)	unsaturated PLE	Lognormal	−0.022	(−0.032, −0.011)	0.000	0.048	36.107
PC (p-34:1)/PC (o-34:2)	unsaturated PLE	Lognormal	−0.025	(−0.038, −0.012)	0.000	0.054	26.013
PC (p-36:1)/PC (o-36:2)	unsaturated PLE	Lognormal	−0.067	(−0.106, −0.029)	0.001	0.064	5.458
PC (p-34:2)/PC (o-34:3)—ESI(−)	unsaturated PLE	Lognormal	−0.025	(−0.038, −0.011)	0.001	0.054	7.542
PC (p-36:4)/PC (o-36:5)—ESI(−)	unsaturated PLE	Lognormal	−0.025	(−0.040, −0.011)	0.001	0.064	7.044
PC (p-34:1)/PC (o-34:2) A	unsaturated PLE	Lognormal	−0.035	(−0.055, −0.015)	0.001	0.064	6.285
**Metabolite**	**Enrichment** **Cluster**	**Regression Model**	**ME/CFS without sr-IBS vs. Control without sr-IBS**				
			**Estimated Coefficient**	**95% CI**	***p*** **-Value**	**FDR**	**Bayes Factor**
Complex Lipids (CL)							
PE (p-36:2)/PE (o-36:3)—ESI(+)	plasmalogens	Lognormal	−0.029	(−0.045, −0.013)	0.000	0.081	6.915
PC (36:2)	unsaturated PC	Lognormal	−0.008	(−0.012, −0.004)	0.000	0.076	8.626
PC (p-34:2)/PC (o-34:3)—ESI(+)	unsaturated PLE	Lognormal	−0.019	(−0.028, −0.010)	0.000	0.076	16.961
PC (p-34:1)/PC (o-34:2)	unsaturated PLE	Lognormal	−0.022	(−0.033, −0.010)	0.000	0.076	30.007

ME/CFS: myalgic encephalomyelitis/chronic fatigue syndrome; sr-IBS: self-reported irritable bowel syndrome; LPC: lysophophatidycholines; PC: phosphatidycholines; PLE: phospholipid ethers; CI, confidence interval; FDR, false discovery rate adjusted *p*-value. For ME/CFS vs. controls, regression models were adjusted for age, sex, race/ethnicity, geographic/clinical site, season of sampling, body mass index, sr-IBS. In the sex-stratified comparisons, regression models were not adjusted for sex. In comparisons within subjects without sr-IBS, regression models were not adjusted for sr-IBS. For lognormal regression, estimated coefficients are interpreted as the differences in the mean values of log-log transformation of metabolite levels between cases and controls. For Gamma regression, estimated coefficients were interpreted as the log of fold change between two groups. Estimations in bold are significant in the corresponding comparisons. We considered a metabolite to be associated with ME/CFS if it satisfied: (1) FDR adjusted *p*-value from regression model < 0.15; (2) Bayes Factor > 3, and (3) 95% highest density credible intervals not covering 0. The credible intervals were extremely similar to the confidence intervals and are shown in Supplementary Tables S2, S4 and S6. No primary metabolites were found to be significantly associated with ME/CFS.

## Data Availability

All data associated with this study are present in the paper or the Supplementary Materials.

## Supplementary Materials

The following supporting information can be downloaded at: https://www.mdpi.com/article/10.3390/ijms23147906/s1.

## Abbreviations

α-KG: alpha-ketoglutarate; β2AdR: β2 Adrenergic receptor; AC: acetylcholine; AdaLasso: adaptive Lasso; AUC: Area under the Receiver Operating Characteristic curve; BA: biogenic amines; BF: Bayes factors; BIC: Bayesian information criterion; BMA: Bayesian Model Average; BMI: body mass index; CDP-choline: cytidine-5′-diphosphocholine; CI: confidence interval; CL: complex lipids; CSH-QTOF MS: quadrupole time-of-flight mass spectrometry; CV: coefficients of variation; DAG: diacylglycerol; DHA: docosahexanoic acid; DSQ: DePaul Symptom Questionnaire; EpODE: ePoxy fatty acids; FDR: false discovery rate; GC-ToF MS: gas chromatography/time-of-flight mass spectrometry; GI: gastrointestinal; GPCR: G protein coupled receptors; HDI: highest density credible interval; HEPE: hydroxy-eicosapentaenoic acid; HILIC-QTOF-MS: hydrophilic interaction liquid chromatography/quadrupole time-of-flight mass spectrometry; Lasso: least absolute shrinkage and selection operator; LC: liquid chromatography; LPC: lysophophatidycholines; M3AChR: M3 Acetylcholine receptor; ME/CFS: myalgic encephalomyelitis/chronic fatigue syndrome; MFI: Multidimensional Fatigue Inventory; MS: mass spectrometry-based assays; OL: oxylipins; PASC: post-acute sequelae of SARS-CoV-2 infection; PCA: principal component analysis; PC: phosphatidycholines; PLE: phospholipid ethers; PGD2: prostaglandin D2; PGF2α: prostaglandin F2α; PM: primary metabolites; PSQI: Pittsburgh Sleep Quality Index; RF: Random Forests; ROC: Receiving Operating Characteristic; ROS: reactive oxygen species; SAM: sorting and assembly machinery; SD: standard deviation; SF-36: Short Form 36 Health Survey; SM: sphingomyelin; sr-IBS: self-reported irritable bowel syndrome; TCA: tricarboxylic acid; TG: triglycerides; TMAO: trimethylamine N-oxide.
