# Supplementary material for: Metabolomic Evidence for Peroxisomal Dysfunction in Myalgic Encephalomyelitis/Chronic Fatigue Syndrome
Source: Int J Mol Sci. 2022 Jul 18;23(14):7906. doi: 10.3390/ijms23147906 (PMC9320121; doi:10.3390/ijms23147906)
Supplement: Supplementary file 1 [file ijms-23-07906-s001.zip › Supplementary Materials_Questionnaire.pdf]

Microbial Discovery and Immunity in ME/CFS - Assessment 1

Project Home | Project Setup | Codebook

Data Dictionary Codebook

02/24/2019 11:44am

Collapse all instruments

	#	Variable / Field Name	Field Label<br>*Field Note*	Field Attributes (Field Type, Validation, Choices, Calculations, etc.)
Instrument: **Cfs Symptom Questionnaire** (cfs_symptom_questionnaire) Collapse				
	1	participant_id	Participant ID	text
	2	subject_id	Please enter your participant/subject ID.	text, Required
	3	me_cfs_1	Do you have ME/CFS?	yesno, Required, Identifier<br>1 Yes<br>0 No
	4	cfs_instructions_1<br><br>Show the field ONLY if:<br>[me_cfs_1] = '1'	Put a check in the box if you had this symptom before CFS. Select the number for how often and how much each symptom has bothered you over the PAST 6 MONTHS. Please read the symptom by the number first, and respond to the three questions that follow for each symptom.	descriptive
	5	fatigue_b4_cfs_1<br><br>Show the field ONLY if:<br>[me_cfs_1] = '1'	1. Fatigue/extreme tiredness: Did you have this symptom BEFORE CFS?	yesno, Required<br>1 Yes<br>0 No
	6	fatigue_freq_1<br><br>Show the field ONLY if:<br>[me_cfs_1] = '1'	Over the PAST 6 MONTHS, how OFTEN have you had this symptom?: Fatigue/extreme tiredness	radio, Required<br>1 0 = none of the time<br>2 1 = a little of the time<br>3 2 = about half the time<br>4 3 = most of the time<br>5 4 = all of the time
	7	fatigue_severe_1<br><br>Show the field ONLY if:<br>[me_cfs_1] = '1'	Over the PAST 6 MONTHS, how MUCH has this symptom bothered you?: Fatigue/extreme tiredness	radio, Required<br>1 0 = symptom not present<br>2 1 = mild<br>3 2 = moderate<br>4 3 = severe<br>5 4 = very severe
	8	feel_dead_b4_cfs_1<br><br>Show the field ONLY if:<br>[me_cfs_1] = '1'	2. Dead, heavy feeling after starting to exercise: Did you have this symptom BEFORE CFS?	yesno, Required<br>1 Yes<br>0 No

	9	feel_dead_freq_1<br>Show the field ONLY if:<br>[me_cfs_1] = '1'	Over the PAST 6 MONTHS, how OFTEN have you had this symptom?: Dead, heavy feeling after starting to exercise	radio, Required<br>1 0 = none of the time<br>2 1 = a little of the time<br>3 2 = about half the time<br>4 3 = most of the time<br>5 4 = all of the time
	10	feel_dead_severe_1<br>Show the field ONLY if:<br>[me_cfs_1] = '1'	Over the PAST 6 MONTHS, how MUCH has this symptom bothered you?: Dead, heavy feeling after starting to exercise	radio, Required<br>1 0 = symptom not present<br>2 1 = mild<br>3 2 = moderate<br>4 3 = severe<br>5 4 = very severe
	11	sore_after_b4_cfs_1<br>Show the field ONLY if:<br>[me_cfs_1] = '1'	3. Next day soreness or fatigue after non-strenuous, everyday activities: Did you have this symptom BEFORE CFS?	yesno, Required<br>1 Yes<br>0 No
	12	sore_after_freq_1<br>Show the field ONLY if:<br>[me_cfs_1] = '1'	Over the PAST 6 MONTHS, how OFTEN have you had this symptom?: Next day soreness or fatigue after non-strenuous, everyday activities	radio, Required<br>1 0 = none of the time<br>2 1 = a little of the time<br>3 2 = about half the time<br>4 3 = most of the time<br>5 4 = all of the time
	13	sore_after_severe_1<br>Show the field ONLY if:<br>[me_cfs_1] = '1'	Over the PAST 6 MONTHS, how MUCH has this symptom bothered you?:Next day soreness or fatigue after non-strenuous, everyday activities	radio, Required<br>1 0 = symptom not present<br>2 1 = mild<br>3 2 = moderate<br>4 3 = severe<br>5 4 = very severe
	14	ment_tired_b4_cfs_1<br>Show the field ONLY if:<br>[me_cfs_1] = '1'	4. Mentally tired after the slightest effort: Did you have this symptom BEFORE CFS?	yesno, Required<br>1 Yes<br>0 No
	15	ment_tired_freq_1<br>Show the field ONLY if:<br>[me_cfs_1] = '1'	Over the PAST 6 MONTHS, how OFTEN have you had this symptom?: Mentally tired after the slightest effort	radio, Required<br>1 0 = none of the time<br>2 1 = a little of the time<br>3 2 = about half the time<br>4 3 = most of the time<br>5 4 = all of the time
	16	ment_tired_severe_2<br>Show the field ONLY if:<br>[me_cfs_1] = '1'	Over the PAST 6 MONTHS, how MUCH has this symptom bothered you?:Mentally tired after the slightest effort	radio, Required<br>1 0 = symptom not present<br>2 1 = mild<br>3 2 = moderate<br>4 3 = severe<br>5 4 = very severe

	17	tired_phys_b4_cfs_1<br>Show the field ONLY if:<br>[me_cfs_1] = '1'	5. Minimum exercise makes you physically tired: Did you have this symptom BEFORE CFS?	yesno, Required<br>1 Yes<br>0 No
	18	tired_phys_freq_1<br>Show the field ONLY if:<br>[me_cfs_1] = '1'	Over the PAST 6 MONTHS, how OFTEN have you had this symptom?: Minimum exercise makes you physically tired	radio, Required<br>1 0 = none of the time<br>2 1 = a little of the time<br>3 2 = about half the time<br>4 3 = most of the time<br>5 4 = all of the time
	19	tired_phys_severe_1<br>Show the field ONLY if:<br>[me_cfs_1] = '1'	Over the PAST 6 MONTHS, how MUCH has this symptom bothered you?: Minimum exercise makes you physically tired	radio, Required<br>1 0 = symptom not present<br>2 1 = mild<br>3 2 = moderate<br>4 3 = severe<br>5 4 = very severe
	20	drain_sick_b4_cfs_1<br>Show the field ONLY if:<br>[me_cfs_1] = '1'	6. Physically drained or sick after mild activity: Did you have this symptom BEFORE CFS?	yesno, Required<br>1 Yes<br>0 No
	21	drain_sick_freq_1<br>Show the field ONLY if:<br>[me_cfs_1] = '1'	Over the PAST 6 MONTHS, how OFTEN have you had this symptom?: Physically drained or sick after mild activity	radio, Required<br>1 0 = none of the time<br>2 1 = a little of the time<br>3 2 = about half the time<br>4 3 = most of the time<br>5 4 = all of the time
	22	drain_sick_severe_1<br>Show the field ONLY if:<br>[me_cfs_1] = '1'	Over the PAST 6 MONTHS, how MUCH has this symptom bothered you?: Physically drained or sick after mild activity	radio, Required<br>1 0 = symptom not present<br>2 1 = mild<br>3 2 = moderate<br>4 3 = severe<br>5 4 = very severe
	23	unrefreshed_b4_cfs_1<br>Show the field ONLY if:<br>[me_cfs_1] = '1'	7. Feeling unrefreshed after you wake up in the morning: Did you have this symptom BEFORE CFS?	yesno, Required<br>1 Yes<br>0 No
	24	unrefreshed_freq_1<br>Show the field ONLY if:<br>[me_cfs_1] = '1'	Over the PAST 6 MONTHS, how OFTEN have you had this symptom?: Feeling unrefreshed after you wake up in the morning	radio, Required<br>1 0 = none of the time<br>2 1 = a little of the time<br>3 2 = about half the time<br>4 3 = most of the time<br>5 4 = all of the time

	25	unrefreshed_severe_1<br><br>Show the field ONLY if:<br>[me_cfs_1] = '1'	Over the PAST 6 MONTHS, how MUCH has this symptom bothered you?: Feeling unrefreshed after you wake up in the morning	radio, Required<br>1 | 0 = symptom not present<br>2 | 1 = mild<br>3 | 2 = moderate<br>4 | 3 = severe<br>5 | 4 = very severe
	26	need_nap_b4_cfs_1<br><br>Show the field ONLY if:<br>[me_cfs_1] = '1'	8. Need to nap daily: Did you have this symptom BEFORE CFS?	yesno, Required<br>1 | Yes<br>0 | No
	27	need_nap_freq_1<br><br>Show the field ONLY if:<br>[me_cfs_1] = '1'	Over the PAST 6 MONTHS, how OFTEN have you had this symptom?: Need to nap daily	radio, Required<br>1 | 0 = none of the time<br>2 | 1 = a little of the time<br>3 | 2 = about half the time<br>4 | 3 = most of the time<br>5 | 4 = all of the time
	28	need_nap_severe_1<br><br>Show the field ONLY if:<br>[me_cfs_1] = '1'	Over the PAST 6 MONTHS, how MUCH has this symptom bothered you?: Need to nap daily	radio, Required<br>1 | 0 = symptom not present<br>2 | 1 = mild<br>3 | 2 = moderate<br>4 | 3 = severe<br>5 | 4 = very severe
	29	fall_asleep_b4_cfs_1<br><br>Show the field ONLY if:<br>[me_cfs_1] = '1'	9. Problems falling asleep: Did you have this symptom BEFORE CFS?	yesno, Required<br>1 | Yes<br>0 | No
	30	fall_asleep_freq_1<br><br>Show the field ONLY if:<br>[me_cfs_1] = '1'	Over the PAST 6 MONTHS, how OFTEN have you had this symptom?: Problems falling asleep	radio, Required<br>1 | 0 = none of the time<br>2 | 1 = a little of the time<br>3 | 2 = about half the time<br>4 | 3 = most of the time<br>5 | 4 = all of the time
	31	fall_asleep_severe_1<br><br>Show the field ONLY if:<br>[me_cfs_1] = '1'	Over the PAST 6 MONTHS, how MUCH has this symptom bothered you?: Problems falling asleep	radio, Required<br>1 | 0 = symptom not present<br>2 | 1 = mild<br>3 | 2 = moderate<br>4 | 3 = severe<br>5 | 4 = very severe
	32	stay_asleep_b4_cfs_1<br><br>Show the field ONLY if:<br>[me_cfs_1] = '1'	10. Problems staying asleep: Did you have this symptom BEFORE CFS?	yesno, Required<br>1 | Yes<br>0 | No

	33	stay_asleep_freq_1<br><br>Show the field ON LY if:<br>[me_cfs_1] = '1'	Over the PAST 6 MONTHS, how OFTEN have you had this symptom?: Problems staying asleep	radio, Required<br>1 | 0 = none of the time<br>2 | 1 = a little of the time<br>3 | 2 = about half the time<br>4 | 3 = most of the time<br>5 | 4 = all of the time
	34	stay_asleep_severe_1<br><br>Show the field ON LY if:<br>[me_cfs_1] = '1'	Over the PAST 6 MONTHS, how MUCH has this symptom bothered you?: Problems staying asleep	radio, Required<br>1 | 0 = symptom not present<br>2 | 1 = mild<br>3 | 2 = moderate<br>4 | 3 = severe<br>5 | 4 = very severe
	35	up_early_b4_cfs_1<br><br>Show the field ON LY if:<br>[me_cfs_1] = '1'	11. Waking up early in the morning (e.g. 3am): Did you have this symptom BEFORE CFS?	yesno, Required<br>1 | Yes<br>0 | No
	36	up_early_freq_1<br><br>Show the field ON LY if:<br>[me_cfs_1] = '1'	Over the PAST 6 MONTHS, how OFTEN have you had this symptom?: Waking up early in the morning (e.g. 3am)	radio, Required<br>1 | 0 = none of the time<br>2 | 1 = a little of the time<br>3 | 2 = about half the time<br>4 | 3 = most of the time<br>5 | 4 = all of the time
	37	up_early_severe_1<br><br>Show the field ON LY if:<br>[me_cfs_1] = '1'	Over the PAST 6 MONTHS, how MUCH has this symptom bothered you?: Waking up early in the morning (e.g. 3am)	radio, Required<br>1 | 0 = symptom not present<br>2 | 1 = mild<br>3 | 2 = moderate<br>4 | 3 = severe<br>5 | 4 = very severe
	38	day_sleep_b4_cfs_1<br><br>Show the field ON LY if:<br>[me_cfs_1] = '1'	12. Sleep all day and stay awake all night: Did you have this symptom BEFORE CFS?	yesno, Required<br>1 | Yes<br>0 | No
	39	day_sleep_freq_1<br><br>Show the field ON LY if:<br>[me_cfs_1] = '1'	Over the PAST 6 MONTHS, how OFTEN have you had this symptom?: Sleep all day and stay awake all night	radio, Required<br>1 | 0 = none of the time<br>2 | 1 = a little of the time<br>3 | 2 = about half the time<br>4 | 3 = most of the time<br>5 | 4 = all of the time
	40	day_sleep_severe_1<br><br>Show the field ON LY if:<br>[me_cfs_1] = '1'	Over the PAST 6 MONTHS, how MUCH has this symptom bothered you?: Sleep all day and stay awake all night	radio, Required<br>1 | 0 = symptom not present<br>2 | 1 = mild<br>3 | 2 = moderate<br>4 | 3 = severe<br>5 | 4 = very severe
	41	musc_pain_b4_cfs_1<br><br>Show the field ON LY if:<br>[me_cfs_1] = '1'	13. Pain or aching in your muscles: Did you have this symptom BEFORE CFS?	yesno, Required<br>1 | Yes<br>0 | No

	42	musc_pain_freq_1<br><br>Show the field ONLY if:<br>[me_cfs_1] = '1'	Over the PAST 6 MONTHS, how OFTEN have you had this symptom?: Pain or aching in your muscles	radio, Required<br>1 0 = none of the time<br>2 1 = a little of the time<br>3 2 = about half the time<br>4 3 = most of the time<br>5 4 = all of the time
	43	musc_pain_severe_1<br><br>Show the field ONLY if:<br>[me_cfs_1] = '1'	Over the PAST 6 MONTHS, how MUCH has this symptom bothered you?: Pain or aching in your muscles	radio, Required<br>1 0 = symptom not present<br>2 1 = mild<br>3 2 = moderate<br>4 3 = severe<br>5 4 = very severe
	44	joint_pain_b4_cfs_1<br><br>Show the field ONLY if:<br>[me_cfs_1] = '1'	14. Pain/stiffness/tenderness in more than one joint without swelling or redness: Did you have this symptom BEFORE CFS?	yesno, Required<br>1 Yes<br>0 No
	45	joint_pain_freq_1<br><br>Show the field ONLY if:<br>[me_cfs_1] = '1'	Over the PAST 6 MONTHS, how OFTEN have you had this symptom?: Pain/stiffness/tenderness in more than one joint without swelling or redness	radio, Required<br>1 0 = none of the time<br>2 1 = a little of the time<br>3 2 = about half the time<br>4 3 = most of the time<br>5 4 = all of the time
	46	joint_pain_severe_1<br><br>Show the field ONLY if:<br>[me_cfs_1] = '1'	Over the PAST 6 MONTHS, how MUCH has this symptom bothered you?: Pain/stiffness/tenderness in more than one joint without swelling or redness	radio, Required<br>1 0 = symptom not present<br>2 1 = mild<br>3 2 = moderate<br>4 3 = severe<br>5 4 = very severe
	47	eye_pain_b4_cfs_1<br><br>Show the field ONLY if:<br>[me_cfs_1] = '1'	15. Eye pain: Did you have this symptom BEFORE CFS?	yesno, Required<br>1 Yes<br>0 No
	48	eye_pain_freq_1<br><br>Show the field ONLY if:<br>[me_cfs_1] = '1'	Over the PAST 6 MONTHS, how OFTEN have you had this symptom?: Eye pain	radio, Required<br>1 0 = none of the time<br>2 1 = a little of the time<br>3 2 = about half the time<br>4 3 = most of the time<br>5 4 = all of the time
	49	eye_pain_severe_1<br><br>Show the field ONLY if:<br>[me_cfs_1] = '1'	Over the PAST 6 MONTHS, how MUCH has this symptom bothered you?: Eye pain	radio, Required<br>1 0 = symptom not present<br>2 1 = mild<br>3 2 = moderate<br>4 3 = severe<br>5 4 = very severe
	50	chest_pain_b4_cfs_1<br><br>Show the field ONLY if:<br>[me_cfs_1] = '1'	16. Chest pain: Did you have this symptom BEFORE CFS?	yesno, Required<br>1 Yes<br>0 No

	51	chest_pain_freq_1<br><br>Show the field ONLY if:<br>[me_cfs_1] = '1'	Over the PAST 6 MONTHS, how OFTEN have you had this symptom?: Chest pain	radio, Required<br>1 | 0 = none of the time<br>2 | 1 = a little of the time<br>3 | 2 = about half the time<br>4 | 3 = most of the time<br>5 | 4 = all of the time
	52	chest_pain_severe_1<br><br>Show the field ONLY if:<br>[me_cfs_1] = '1'	Over the PAST 6 MONTHS, how MUCH has this symptom bothered you?: Chest pain	radio, Required<br>1 | 0 = symptom not present<br>2 | 1 = mild<br>3 | 2 = moderate<br>4 | 3 = severe<br>5 | 4 = very severe
	53	bloating_b4_cfs_1<br><br>Show the field ONLY if:<br>[me_cfs_1] = '1'	17. Bloating: Did you have this symptom BEFORE CFS?	yesno, Required<br>1 | Yes<br>0 | No
	54	bloating_freq_1<br><br>Show the field ONLY if:<br>[me_cfs_1] = '1'	Over the PAST 6 MONTHS, how OFTEN have you had this symptom?: Bloating	radio, Required<br>1 | 0 = none of the time<br>2 | 1 = a little of the time<br>3 | 2 = about half the time<br>4 | 3 = most of the time<br>5 | 4 = all of the time
	55	bloating_severe_1<br><br>Show the field ONLY if:<br>[me_cfs_1] = '1'	Over the PAST 6 MONTHS, how MUCH has this symptom bothered you?: Bloating	radio, Required<br>1 | 0 = symptom not present<br>2 | 1 = mild<br>3 | 2 = moderate<br>4 | 3 = severe<br>5 | 4 = very severe
	56	ab_pain_b4_cfs_1<br><br>Show the field ONLY if:<br>[me_cfs_1] = '1'	18. Abdomen/stomach pain: Did you have this symptom BEFORE CFS?	yesno, Required<br>1 | Yes<br>0 | No
	57	ab_pain_freq_1<br><br>Show the field ONLY if:<br>[me_cfs_1] = '1'	Over the PAST 6 MONTHS, how OFTEN have you had this symptom?: Abdomen/stomach pain	radio, Required<br>1 | 0 = none of the time<br>2 | 1 = a little of the time<br>3 | 2 = about half the time<br>4 | 3 = most of the time<br>5 | 4 = all of the time
	58	ab_pain_severe_1<br><br>Show the field ONLY if:<br>[me_cfs_1] = '1'	Over the PAST 6 MONTHS, how MUCH has this symptom bothered you?: Abdomen/stomach pain	radio, Required<br>1 | 0 = symptom not present<br>2 | 1 = mild<br>3 | 2 = moderate<br>4 | 3 = severe<br>5 | 4 = very severe
	59	headache_b4_cfs_1<br><br>Show the field ONLY if:<br>[me_cfs_1] = '1'	19. Headaches: Did you have this symptom BEFORE CFS?	yesno, Required<br>1 | Yes<br>0 | No

	60	headache_freq_1<br><br>Show the field ONLY if:<br>[me_cfs_1] = '1'	Over the PAST 6 MONTHS, how OFTEN have you had this symptom?: Headaches	radio, Required<br>1 | 0 = none of the time<br>2 | 1 = a little of the time<br>3 | 2 = about half the time<br>4 | 3 = most of the time<br>5 | 4 = all of the time
	61	headache_severe_1<br><br>Show the field ONLY if:<br>[me_cfs_1] = '1'	Over the PAST 6 MONTHS, how MUCH has this symptom bothered you?: Headaches	radio, Required<br>1 | 0 = symptom not present<br>2 | 1 = mild<br>3 | 2 = moderate<br>4 | 3 = severe<br>5 | 4 = very severe
	62	musc_twitch_b4_cfs_1<br><br>Show the field ONLY if:<br>[me_cfs_1] = '1'	20. Muscle twitches: Did you have this symptom BEFORE CFS?	yesno, Required<br>1 | Yes<br>0 | No
	63	musc_twitch_freq_1<br><br>Show the field ONLY if:<br>[me_cfs_1] = '1'	Over the PAST 6 MONTHS, how OFTEN have you had this symptom?: Muscle twitches	radio, Required<br>1 | 0 = none of the time<br>2 | 1 = a little of the time<br>3 | 2 = about half the time<br>4 | 3 = most of the time<br>5 | 4 = all of the time
	64	musc_twitch_severe_1<br><br>Show the field ONLY if:<br>[me_cfs_1] = '1'	Over the PAST 6 MONTHS, how MUCH has this symptom bothered you?: Muscle twitches	radio, Required<br>1 | 0 = symptom not present<br>2 | 1 = mild<br>3 | 2 = moderate<br>4 | 3 = severe<br>5 | 4 = very severe
	65	weak_musc_b4_cfs_1<br><br>Show the field ONLY if:<br>[me_cfs_1] = '1'	21. Muscle weakness: Did you have this symptom BEFORE CFS?	yesno, Required<br>1 | Yes<br>0 | No
	66	weak_musc_freq_1<br><br>Show the field ONLY if:<br>[me_cfs_1] = '1'	Over the PAST 6 MONTHS, how OFTEN have you had this symptom?: Muscle weakness	radio, Required<br>1 | 0 = none of the time<br>2 | 1 = a little of the time<br>3 | 2 = about half the time<br>4 | 3 = most of the time<br>5 | 4 = all of the time
	67	weak_musc_severe_1<br><br>Show the field ONLY if:<br>[me_cfs_1] = '1'	Over the PAST 6 MONTHS, how MUCH has this symptom bothered you?: Muscle weakness	radio, Required<br>1 | 0 = symptom not present<br>2 | 1 = mild<br>3 | 2 = moderate<br>4 | 3 = severe<br>5 | 4 = very severe

	68	sens2noise_b4_cfs_1<br>Show the field ONLY if:<br>[me_cfs_1] = '1'	22. Sensitivity to noise: Did you have this symptom BEFORE CFS?	yesno, Required<br>1 Yes<br>0 No
	69	sens2noise_freq_1<br>Show the field ONLY if:<br>[me_cfs_1] = '1'	Over the PAST 6 MONTHS, how OFTEN have you had this symptom?: Sensitivity to noise	radio, Required<br>1 0 = none of the time<br>2 1 = a little of the time<br>3 2 = about half the time<br>4 3 = most of the time<br>5 4 = all of the time
	70	sens2noise_severe_1<br>Show the field ONLY if:<br>[me_cfs_1] = '1'	Over the PAST 6 MONTHS, how MUCH has this symptom bothered you?: Sensitivity to noise	radio, Required<br>1 0 = symptom not present<br>2 1 = mild<br>3 2 = moderate<br>4 3 = severe<br>5 4 = very severe
	71	sens2light_b4_cfs_1<br>Show the field ONLY if:<br>[me_cfs_1] = '1'	23. Sensitivity to bright lights: Did you have this symptom BEFORE CFS?	yesno, Required<br>1 Yes<br>0 No
	72	sens2light_freq_1<br>Show the field ONLY if:<br>[me_cfs_1] = '1'	Over the PAST 6 MONTHS, how OFTEN have you had this symptom?: Sensitivity to bright lights	radio, Required<br>1 0 = none of the time<br>2 1 = a little of the time<br>3 2 = about half the time<br>4 3 = most of the time<br>5 4 = all of the time
	73	sens2light_severe_1<br>Show the field ONLY if:<br>[me_cfs_1] = '1'	Over the PAST 6 MONTHS, how MUCH has this symptom bothered you?: Sensitivity to bright lights	radio, Required<br>1 0 = symptom not present<br>2 1 = mild<br>3 2 = moderate<br>4 3 = severe<br>5 4 = very severe
	74	mem_probs_b4_cfs_1<br>Show the field ONLY if:<br>[me_cfs_1] = '1'	24. Problems remembering things: Did you have this symptom BEFORE CFS?	yesno, Required<br>1 Yes<br>0 No
	75	mem_probs_freq_1<br>Show the field ONLY if:<br>[me_cfs_1] = '1'	Over the PAST 6 MONTHS, how OFTEN have you had this symptom?: Problems remembering things	radio, Required<br>1 0 = none of the time<br>2 1 = a little of the time<br>3 2 = about half the time<br>4 3 = most of the time<br>5 4 = all of the time

	76	mem_probs_severe_1<br><br>Show the field ONLY if:<br>[me_cfs_1] = '1'	Over the PAST 6 MONTHS, how MUCH has this symptom bothered you?: Problems remembering things	radio, Required<br>1 | 0 = symptom not present<br>2 | 1 = mild<br>3 | 2 = moderate<br>4 | 3 = severe<br>5 | 4 = very severe
	77	no_attent_b4_cfs_1<br><br>Show the field ONLY if:<br>[me_cfs_1] = '1'	25. Difficulty paying attention: Did you have this symptom BEFORE CFS?	yesno, Required<br>1 | Yes<br>0 | No
	78	no_attent_freq_1<br><br>Show the field ONLY if:<br>[me_cfs_1] = '1'	Over the PAST 6 MONTHS, how OFTEN have you had this symptom?: Difficulty paying attention	radio, Required<br>1 | 0 = none of the time<br>2 | 1 = a little of the time<br>3 | 2 = about half the time<br>4 | 3 = most of the time<br>5 | 4 = all of the time
	79	no_attent_severe_1<br><br>Show the field ONLY if:<br>[me_cfs_1] = '1'	Over the PAST 6 MONTHS, how MUCH has this symptom bothered you?: Difficulty paying attention	radio, Required<br>1 | 0 = symptom not present<br>2 | 1 = mild<br>3 | 2 = moderate<br>4 | 3 = severe<br>5 | 4 = very severe
	80	word_probs_b4_cfs_1<br><br>Show the field ONLY if:<br>[me_cfs_1] = '1'	26. Difficulty finding the right word to say or expressing thoughts: Did you have this symptom BEFORE CFS?	yesno, Required<br>1 | Yes<br>0 | No
	81	word_probs_freq_1<br><br>Show the field ONLY if:<br>[me_cfs_1] = '1'	Over the PAST 6 MONTHS, how OFTEN have you had this symptom?: Difficulty finding the right word to say or expressing thoughts	radio, Required<br>1 | 0 = none of the time<br>2 | 1 = a little of the time<br>3 | 2 = about half the time<br>4 | 3 = most of the time<br>5 | 4 = all of the time
	82	word_probs_severe_1<br><br>Show the field ONLY if:<br>[me_cfs_1] = '1'	Over the PAST 6 MONTHS, how MUCH has this symptom bothered you?: Difficulty finding the right word to say or expressing thoughts	radio, Required<br>1 | 0 = symptom not present<br>2 | 1 = mild<br>3 | 2 = moderate<br>4 | 3 = severe<br>5 | 4 = very severe
	83	no_comp_b4_cfs_1<br><br>Show the field ONLY if:<br>[me_cfs_1] = '1'	27. Difficulty understanding things: Did you have this symptom BEFORE CFS?	yesno, Required<br>1 | Yes<br>0 | No
	84	no_comp_freq_1<br><br>Show the field ONLY if:<br>[me_cfs_1] = '1'	Over the PAST 6 MONTHS, how OFTEN have you had this symptom?: Difficulty understanding things	radio, Required<br>1 | 0 = none of the time<br>2 | 1 = a little of the time<br>3 | 2 = about half the time<br>4 | 3 = most of the time<br>5 | 4 = all of the time

	85	no_comp_severe_1<br><br>Show the field ONLY if:<br>[me_cfs_1] = '1'	Over the PAST 6 MONTHS, how MUCH has this symptom bothered you?: Difficulty understanding things	radio, Required<br>1 | 0 = symptom not present<br>2 | 1 = mild<br>3 | 2 = moderate<br>4 | 3 = severe<br>5 | 4 = very severe
	86	focus_1thing_b4_cfs_1<br><br>Show the field ONLY if:<br>[me_cfs_1] = '1'	28. Only can focus on one thing at a time: Did you have this symptom BEFORE CFS?	yesno, Required<br>1 | Yes<br>0 | No
	87	focus_1thing_freq_1<br><br>Show the field ONLY if:<br>[me_cfs_1] = '1'	Over the PAST 6 MONTHS, how OFTEN have you had this symptom?: Only can focus on one thing at a time	radio, Required<br>1 | 0 = none of the time<br>2 | 1 = a little of the time<br>3 | 2 = about half the time<br>4 | 3 = most of the time<br>5 | 4 = all of the time
	88	focus_1thing_severe_1<br><br>Show the field ONLY if:<br>[me_cfs_1] = '1'	Over the PAST 6 MONTHS, how MUCH has this symptom bothered you?: Only can focus on one thing at a time	radio, Required<br>1 | 0 = symptom not present<br>2 | 1 = mild<br>3 | 2 = moderate<br>4 | 3 = severe<br>5 | 4 = very severe
	89	focus_eye_b4_cfs_1<br><br>Show the field ONLY if:<br>[me_cfs_1] = '1'	29. Unable to focus vision and attention: Did you have this symptom BEFORE CFS?	yesno, Required<br>1 | Yes<br>0 | No
	90	focus_eye_freq_1<br><br>Show the field ONLY if:<br>[me_cfs_1] = '1'	Over the PAST 6 MONTHS, how OFTEN have you had this symptom?: Unable to focus vision and attention	radio, Required<br>1 | 0 = none of the time<br>2 | 1 = a little of the time<br>3 | 2 = about half the time<br>4 | 3 = most of the time<br>5 | 4 = all of the time
	91	focus_eye_severe_1<br><br>Show the field ONLY if:<br>[me_cfs_1] = '1'	Over the PAST 6 MONTHS, how MUCH has this symptom bothered you?: Unable to focus vision and attention	radio, Required<br>1 | 0 = symptom not present<br>2 | 1 = mild<br>3 | 2 = moderate<br>4 | 3 = severe<br>5 | 4 = very severe
	92	no_depth_b4_cfs_1<br><br>Show the field ONLY if:<br>[me_cfs_1] = '1'	30. Loss of depth perception: Did you have this symptom BEFORE CFS?	yesno, Required<br>1 | Yes<br>0 | No
	93	no_depth_freq_1<br><br>Show the field ONLY if:<br>[me_cfs_1] = '1'	Over the PAST 6 MONTHS, how OFTEN have you had this symptom?: Loss of depth perception	radio, Required<br>1 | 0 = none of the time<br>2 | 1 = a little of the time<br>3 | 2 = about half the time<br>4 | 3 = most of the time<br>5 | 4 = all of the time

	94	no_depth_severe_1<br>Show the field ONLY if:<br>[me_cfs_1] = '1'	Over the PAST 6 MONTHS, how MUCH has this symptom bothered you?: Loss of depth perception	radio, Required<br>1 | 0 = symptom not present<br>2 | 1 = mild<br>3 | 2 = moderate<br>4 | 3 = severe<br>5 | 4 = very severe
	95	slo_thought_b4_cfs_1<br>Show the field ONLY if:<br>[me_cfs_1] = '1'	31. Slowness of thought: Did you have this symptom BEFORE CFS?	yesno, Required<br>1 | Yes<br>0 | No
	96	slo_thought_freq_1<br>Show the field ONLY if:<br>[me_cfs_1] = '1'	Over the PAST 6 MONTHS, how OFTEN have you had this symptom?:Slowness of thought	radio, Required<br>1 | 0 = none of the time<br>2 | 1 = a little of the time<br>3 | 2 = about half the time<br>4 | 3 = most of the time<br>5 | 4 = all of the time
	97	slo_thought_severe_1<br>Show the field ONLY if:<br>[me_cfs_1] = '1'	Over the PAST 6 MONTHS, how MUCH has this symptom bothered you?:Slowness of thought	radio, Required<br>1 | 0 = symptom not present<br>2 | 1 = mild<br>3 | 2 = moderate<br>4 | 3 = severe<br>5 | 4 = very severe
	98	forgetful_b4_cfs_1<br>Show the field ONLY if:<br>[me_cfs_1] = '1'	32. Absent-mindedness or forgetfulness: Did you have this symptom BEFORE CFS?	yesno, Required<br>1 | Yes<br>0 | No
	99	forgetful_freq_1<br>Show the field ONLY if:<br>[me_cfs_1] = '1'	Over the PAST 6 MONTHS, how OFTEN have you had this symptom?: Absent-mindedness or forgetfulness	radio, Required<br>1 | 0 = none of the time<br>2 | 1 = a little of the time<br>3 | 2 = about half the time<br>4 | 3 = most of the time<br>5 | 4 = all of the time
	100	forgetful_severe_1<br>Show the field ONLY if:<br>[me_cfs_1] = '1'	Over the PAST 6 MONTHS, how MUCH has this symptom bothered you?: Absent-mindedness or forgetfulness	radio, Required<br>1 | 0 = symptom not present<br>2 | 1 = mild<br>3 | 2 = moderate<br>4 | 3 = severe<br>5 | 4 = very severe
	101	bladder_b4_cfs_1<br>Show the field ONLY if:<br>[me_cfs_1] = '1'	33. Bladder problems: Did you have this symptom BEFORE CFS?	yesno, Required<br>1 | Yes<br>0 | No
	102	bladder_freq_1<br>Show the field ONLY if:<br>[me_cfs_1] = '1'	Over the PAST 6 MONTHS, how OFTEN have you had this symptom?: Bladder problems	radio, Required<br>1 | 0 = none of the time<br>2 | 1 = a little of the time<br>3 | 2 = about half the time<br>4 | 3 = most of the time<br>5 | 4 = all of the time

	103	bladder_severe_1<br><br>Show the field ONLY if:<br>[me_cfs_1] = '1'	Over the PAST 6 MONTHS, how MUCH has this symptom bothered you?: Bladder problems	radio, Required<br>1 0 = symptom not present<br>2 1 = mild<br>3 2 = moderate<br>4 3 = severe<br>5 4 = very severe
	104	bowel_b4_cfs_1<br><br>Show the field ONLY if:<br>[me_cfs_1] = '1'	34. Irritable bowel problems: Did you have this symptom BEFORE CFS?	yesno, Required<br>1 Yes<br>0 No
	105	bowel_freq_1<br><br>Show the field ONLY if:<br>[me_cfs_1] = '1'	Over the PAST 6 MONTHS, how OFTEN have you had this symptom?: Irritable bowel problems	radio, Required<br>1 0 = none of the time<br>2 1 = a little of the time<br>3 2 = about half the time<br>4 3 = most of the time<br>5 4 = all of the time
	106	bowel_severe_1<br><br>Show the field ONLY if:<br>[me_cfs_1] = '1'	Over the PAST 6 MONTHS, how MUCH has this symptom bothered you?: Irritable bowel problems	radio, Required<br>1 0 = symptom not present<br>2 1 = mild<br>3 2 = moderate<br>4 3 = severe<br>5 4 = very severe
	107	nausea_b4_cfs_1<br><br>Show the field ONLY if:<br>[me_cfs_1] = '1'	35. Nausea: Did you have this symptom BEFORE CFS?	yesno, Required<br>1 Yes<br>0 No
	108	nausea_freq_1<br><br>Show the field ONLY if:<br>[me_cfs_1] = '1'	Over the PAST 6 MONTHS, how OFTEN have you had this symptom?: Nausea	radio, Required<br>1 0 = none of the time<br>2 1 = a little of the time<br>3 2 = about half the time<br>4 3 = most of the time<br>5 4 = all of the time
	109	nausea_severe_1<br><br>Show the field ONLY if:<br>[me_cfs_1] = '1'	Over the PAST 6 MONTHS, how MUCH has this symptom bothered you?: Nausea	radio, Required<br>1 0 = symptom not present<br>2 1 = mild<br>3 2 = moderate<br>4 3 = severe<br>5 4 = very severe
	110	unsteady_b4_cfs_1<br><br>Show the field ONLY if:<br>[me_cfs_1] = '1'	36. Feeling unsteady on your feet, like you might fall: Did you have this symptom BEFORE CFS?	yesno, Required<br>1 Yes<br>0 No
	111	unsteady_freq_1<br><br>Show the field ONLY if:<br>[me_cfs_1] = '1'	Over the PAST 6 MONTHS, how OFTEN have you had this symptom?: Feeling unsteady on your feet, like you might fall	radio, Required<br>1 0 = none of the time<br>2 1 = a little of the time<br>3 2 = about half the time<br>4 3 = most of the time<br>5 4 = all of the time

	112	unsteady_severe_1<br><br>Show the field ONLY if:<br>[me_cfs_1] = '1'	Over the PAST 6 MONTHS, how MUCH has this symptom bothered you?: Feeling unsteady on your feet, like you might fall	radio, Required<br>1 0 = symptom not present<br>2 1 = mild<br>3 2 = moderate<br>4 3 = severe<br>5 4 = very severe
	113	breathing_b4_cfs_1<br><br>Show the field ONLY if:<br>[me_cfs_1] = '1'	37. Shortness of breath or trouble catching your breath: Did you have this symptom BEFORE CFS?	yesno, Required<br>1 Yes<br>0 No
	114	breathing_freq_1<br><br>Show the field ONLY if:<br>[me_cfs_1] = '1'	Over the PAST 6 MONTHS, how OFTEN have you had this symptom?: Shortness of breath or trouble catching your breath	radio, Required<br>1 0 = none of the time<br>2 1 = a little of the time<br>3 2 = about half the time<br>4 3 = most of the time<br>5 4 = all of the time
	115	breathing_severe_1<br><br>Show the field ONLY if:<br>[me_cfs_1] = '1'	Over the PAST 6 MONTHS, how MUCH has this symptom bothered you?: Shortness of breath or trouble catching your breath	radio, Required<br>1 0 = symptom not present<br>2 1 = mild<br>3 2 = moderate<br>4 3 = severe<br>5 4 = very severe
	116	dizzy_b4_cfs_1<br><br>Show the field ONLY if:<br>[me_cfs_1] = '1'	38. Dizziness or fainting: Did you have this symptom BEFORE CFS?	yesno, Required<br>1 Yes<br>0 No
	117	dizzy_freq_1<br><br>Show the field ONLY if:<br>[me_cfs_1] = '1'	Over the PAST 6 MONTHS, how OFTEN have you had this symptom?: Dizziness or fainting	radio, Required<br>1 0 = none of the time<br>2 1 = a little of the time<br>3 2 = about half the time<br>4 3 = most of the time<br>5 4 = all of the time
	118	dizzy_severe_1<br><br>Show the field ONLY if:<br>[me_cfs_1] = '1'	Over the PAST 6 MONTHS, how MUCH has this symptom bothered you?: Dizziness or fainting	radio, Required<br>1 0 = symptom not present<br>2 1 = mild<br>3 2 = moderate<br>4 3 = severe<br>5 4 = very severe
	119	bad_heart_b4_cfs_1<br><br>Show the field ONLY if:<br>[me_cfs_1] = '1'	39. Irregular heart beats: Did you have this symptom BEFORE CFS?	yesno, Required<br>1 Yes<br>0 No
	120	bad_heart_freq_1<br><br>Show the field ONLY if:<br>[me_cfs_1] = '1'	Over the PAST 6 MONTHS, how OFTEN have you had this symptom?: Irregular heart beats	radio, Required<br>1 0 = none of the time<br>2 1 = a little of the time<br>3 2 = about half the time<br>4 3 = most of the time<br>5 4 = all of the time

	121	bad_heart_severe_1<br><br>Show the field ONLY if:<br>[me_cfs_1] = '1'	Over the PAST 6 MONTHS, how MUCH has this symptom bothered you?: Irregular heart beats	radio, Required<br>1 | 0 = symptom not present<br>2 | 1 = mild<br>3 | 2 = moderate<br>4 | 3 = severe<br>5 | 4 = very severe
	122	weight_b4_cfs_1<br><br>Show the field ONLY if:<br>[me_cfs_1] = '1'	40. Losing or gaining weight without trying: Did you have this symptom BEFORE CFS?	yesno, Required<br>1 | Yes<br>0 | No
	123	weight_freq_1<br><br>Show the field ONLY if:<br>[me_cfs_1] = '1'	Over the PAST 6 MONTHS, how OFTEN have you had this symptom?: Losing or gaining weight without trying	radio, Required<br>1 | 0 = none of the time<br>2 | 1 = a little of the time<br>3 | 2 = about half the time<br>4 | 3 = most of the time<br>5 | 4 = all of the time
	124	weight_severe_1<br><br>Show the field ONLY if:<br>[me_cfs_1] = '1'	Over the PAST 6 MONTHS, how MUCH has this symptom bothered you?: Losing or gaining weight without trying	radio, Required<br>1 | 0 = symptom not present<br>2 | 1 = mild<br>3 | 2 = moderate<br>4 | 3 = severe<br>5 | 4 = very severe
	125	appetite_b4_cfs_1<br><br>Show the field ONLY if:<br>[me_cfs_1] = '1'	41. No appetite: Did you have this symptom BEFORE CFS?	yesno, Required<br>1 | Yes<br>0 | No
	126	appetite_freq_1<br><br>Show the field ONLY if:<br>[me_cfs_1] = '1'	Over the PAST 6 MONTHS, how OFTEN have you had this symptom?: No appetite	radio, Required<br>1 | 0 = none of the time<br>2 | 1 = a little of the time<br>3 | 2 = about half the time<br>4 | 3 = most of the time<br>5 | 4 = all of the time
	127	appetite_severe_1<br><br>Show the field ONLY if:<br>[me_cfs_1] = '1'	Over the PAST 6 MONTHS, how MUCH has this symptom bothered you?: No appetite	radio, Required<br>1 | 0 = symptom not present<br>2 | 1 = mild<br>3 | 2 = moderate<br>4 | 3 = severe<br>5 | 4 = very severe
	128	hand_sweat_1<br><br>Show the field ONLY if:<br>[me_cfs_1] = '1'	42. Sweating hands: Did you have this symptom BEFORE CFS?	yesno, Required<br>1 | Yes<br>0 | No
	129	hand_sweat_freq_1<br><br>Show the field ONLY if:<br>[me_cfs_1] = '1'	Over the PAST 6 MONTHS, how OFTEN have you had this symptom?: Sweating hands	radio, Required<br>1 | 0 = none of the time<br>2 | 1 = a little of the time<br>3 | 2 = about half the time<br>4 | 3 = most of the time<br>5 | 4 = all of the time

	130	hand_sweat_severe_1<br><br>Show the field ONLY if:<br>[me_cfs_1] = '1'	Over the PAST 6 MONTHS, how MUCH has this symptom bothered you?: Sweating hands	radio, Required<br>1 0 = symptom not present<br>2 1 = mild<br>3 2 = moderate<br>4 3 = severe<br>5 4 = very severe
	131	night_sweat_b4_cfs_1<br><br>Show the field ONLY if:<br>[me_cfs_1] = '1'	43. Night sweats: Did you have this symptom BEFORE CFS?	yesno, Required<br>1 Yes<br>0 No
	132	night_sweat_freq_1<br><br>Show the field ONLY if:<br>[me_cfs_1] = '1'	Over the PAST 6 MONTHS, how OFTEN have you had this symptom?: Night sweats	radio, Required<br>1 0 = none of the time<br>2 1 = a little of the time<br>3 2 = about half the time<br>4 3 = most of the time<br>5 4 = all of the time
	133	night_sweat_severe_1<br><br>Show the field ONLY if:<br>[me_cfs_1] = '1'	Over the PAST 6 MONTHS, how MUCH has this symptom bothered you?: Night sweats	radio, Required<br>1 0 = symptom not present<br>2 1 = mild<br>3 2 = moderate<br>4 3 = severe<br>5 4 = very severe
	134	cold_limbs_b4_cfs_1<br><br>Show the field ONLY if:<br>[me_cfs_1] = '1'	44. Cold limbs (e.g. arms, legs, hands): Did you have this symptom BEFORE CFS?	yesno, Required<br>1 Yes<br>0 No
	135	cold_limbs_freq_1<br><br>Show the field ONLY if:<br>[me_cfs_1] = '1'	Over the PAST 6 MONTHS, how OFTEN have you had this symptom?: Cold limbs (e.g. arms, legs, hands)	radio, Required<br>1 0 = none of the time<br>2 1 = a little of the time<br>3 2 = about half the time<br>4 3 = most of the time<br>5 4 = all of the time
	136	cold_limbs_severe_1<br><br>Show the field ONLY if:<br>[me_cfs_1] = '1'	Over the PAST 6 MONTHS, how MUCH has this symptom bothered you?: Cold limbs (e.g. arms, legs, hands)	radio, Required<br>1 0 = symptom not present<br>2 1 = mild<br>3 2 = moderate<br>4 3 = severe<br>5 4 = very severe
	137	chills_b4_cfs_1<br><br>Show the field ONLY if:<br>[me_cfs_1] = '1'	45. Feeling chills or shivers: Did you have this symptom BEFORE CFS?	yesno, Required<br>1 Yes<br>0 No
	138	chills_freq_1<br><br>Show the field ONLY if:<br>[me_cfs_1] = '1'	Over the PAST 6 MONTHS, how OFTEN have you had this symptom?: Feeling chills or shivers	radio, Required<br>1 0 = none of the time<br>2 1 = a little of the time<br>3 2 = about half the time<br>4 3 = most of the time<br>5 4 = all of the time

	139	chills_severe_1<br><br>Show the field ONLY if:<br>[me_cfs_1] = '1'	Over the PAST 6 MONTHS, how MUCH has this symptom bothered you?: Feeling chills or shivers	radio, Required<br>1 | 0 = symptom not present<br>2 | 1 = mild<br>3 | 2 = moderate<br>4 | 3 = severe<br>5 | 4 = very severe
	140	hot_cold_b4_cfs_1<br><br>Show the field ONLY if:<br>[me_cfs_1] = '1'	46. Feeling hot or cold for no reason: Did you have this symptom BEFORE CFS?	yesno, Required<br>1 | Yes<br>0 | No
	141	hot_cold_freq_1<br><br>Show the field ONLY if:<br>[me_cfs_1] = '1'	Over the PAST 6 MONTHS, how OFTEN have you had this symptom?: Feeling hot or cold for no reason	radio, Required<br>1 | 0 = none of the time<br>2 | 1 = a little of the time<br>3 | 2 = about half the time<br>4 | 3 = most of the time<br>5 | 4 = all of the time
	142	hot_cold_severe_1<br><br>Show the field ONLY if:<br>[me_cfs_1] = '1'	Over the PAST 6 MONTHS, how MUCH has this symptom bothered you?: Feeling hot or cold for no reason	radio, Required<br>1 | 0 = symptom not present<br>2 | 1 = mild<br>3 | 2 = moderate<br>4 | 3 = severe<br>5 | 4 = very severe
	143	hi_temp_b4_cfs_1<br><br>Show the field ONLY if:<br>[me_cfs_1] = '1'	47. Feeling like you have a high temperature: Did you have this symptom BEFORE CFS?	yesno, Required<br>1 | Yes<br>0 | No
	144	hi_temp_freq_1<br><br>Show the field ONLY if:<br>[me_cfs_1] = '1'	Over the PAST 6 MONTHS, how OFTEN have you had this symptom?: Feeling like you have a high temperature	radio, Required<br>1 | 0 = none of the time<br>2 | 1 = a little of the time<br>3 | 2 = about half the time<br>4 | 3 = most of the time<br>5 | 4 = all of the time
	145	hi_temp_severe_1<br><br>Show the field ONLY if:<br>[me_cfs_1] = '1'	Over the PAST 6 MONTHS, how MUCH has this symptom bothered you?: Feeling like you have a high temperature	radio, Required<br>1 | 0 = symptom not present<br>2 | 1 = mild<br>3 | 2 = moderate<br>4 | 3 = severe<br>5 | 4 = very severe
	146	lo_temp_b4_cfs_1<br><br>Show the field ONLY if:<br>[me_cfs_1] = '1'	48. Feeling like you have a low temperature: Did you have this symptom BEFORE CFS?	yesno, Required<br>1 | Yes<br>0 | No
	147	lo_temp_freq_1<br><br>Show the field ONLY if:<br>[me_cfs_1] = '1'	Over the PAST 6 MONTHS, how OFTEN have you had this symptom?: Feeling like you have a low temperature	radio, Required<br>1 | 0 = none of the time<br>2 | 1 = a little of the time<br>3 | 2 = about half the time<br>4 | 3 = most of the time<br>5 | 4 = all of the time

148	lo_temp_severe_1<br><br>Show the field ONLY if:<br>[me_cfs_1] = '1'	Over the PAST 6 MONTHS, how MUCH has this symptom bothered you?: Feeling like you have a low temperature	radio, Required<br>1 0 = symptom not present<br>2 1 = mild<br>3 2 = moderate<br>4 3 = severe<br>5 4 = very severe
149	no_alcohol_b4_cfs_1<br><br>Show the field ONLY if:<br>[me_cfs_1] = '1'	49. Alcohol intolerance: Did you have this symptom BEFORE CFS?	yesno, Required<br>1 Yes<br>0 No
150	no_alcohol_freq_1<br><br>Show the field ONLY if:<br>[me_cfs_1] = '1'	Over the PAST 6 MONTHS, how OFTEN have you had this symptom?: Alcohol intolerance	radio, Required<br>1 0 = none of the time<br>2 1 = a little of the time<br>3 2 = about half the time<br>4 3 = most of the time<br>5 4 = all of the time
151	no_alcohol_severe_1<br><br>Show the field ONLY if:<br>[me_cfs_1] = '1'	Over the PAST 6 MONTHS, how MUCH has this symptom bothered you?: Alcohol intolerance	radio, Required<br>1 0 = symptom not present<br>2 1 = mild<br>3 2 = moderate<br>4 3 = severe<br>5 4 = very severe
152	sore_throat_b4_cfs_1<br><br>Show the field ONLY if:<br>[me_cfs_1] = '1'	50. Sore throat: Did you have this symptom BEFORE CFS?	yesno, Required<br>1 Yes<br>0 No
153	sore_throat_freq_1<br><br>Show the field ONLY if:<br>[me_cfs_1] = '1'	Over the PAST 6 MONTHS, how OFTEN have you had this symptom?: Sore throat	radio, Required<br>1 0 = none of the time<br>2 1 = a little of the time<br>3 2 = about half the time<br>4 3 = most of the time<br>5 4 = all of the time
154	sore_throat_severe_1<br><br>Show the field ONLY if:<br>[me_cfs_1] = '1'	Over the PAST 6 MONTHS, how MUCH has this symptom bothered you?: Sore throat	radio, Required<br>1 0 = symptom not present<br>2 1 = mild<br>3 2 = moderate<br>4 3 = severe<br>5 4 = very severe
155	lymph_b4_cfs_1<br><br>Show the field ONLY if:<br>[me_cfs_1] = '1'	51. Tender/sore lymph nodes: Did you have this symptom BEFORE CFS?	yesno, Required<br>1 Yes<br>0 No
156	lymph_freq_1<br><br>Show the field ONLY if:<br>[me_cfs_1] = '1'	Over the PAST 6 MONTHS, how OFTEN have you had this symptom?: Tender/sore lymph nodes	radio, Required<br>1 0 = none of the time<br>2 1 = a little of the time<br>3 2 = about half the time<br>4 3 = most of the time<br>5 4 = all of the time

	157 lymph_severe_1<br><br>Show the field ONLY if:<br>[me_cfs_1] = '1'	Over the PAST 6 MONTHS, how MUCH has this symptom bothered you?: Tender/sore lymph nodes	radio, Required<br>1 | 0 = symptom not present<br>2 | 1 = mild<br>3 | 2 = moderate<br>4 | 3 = severe<br>5 | 4 = very severe
	158 fever_b4_cfs_1<br><br>Show the field ONLY if:<br>[me_cfs_1] = '1'	52. Fever: Did you have this symptom BEFORE CFS?	yesno, Required<br>1 | Yes<br>0 | No
	159 fever_freq_1<br><br>Show the field ONLY if:<br>[me_cfs_1] = '1'	Over the PAST 6 MONTHS, how OFTEN have you had this symptom?: Fever	radio, Required<br>1 | 0 = none of the time<br>2 | 1 = a little of the time<br>3 | 2 = about half the time<br>4 | 3 = most of the time<br>5 | 4 = all of the time
	160 fever_severe_1<br><br>Show the field ONLY if:<br>[me_cfs_1] = '1'	Over the PAST 6 MONTHS, how MUCH has this symptom bothered you?: Fever	radio, Required<br>1 | 0 = symptom not present<br>2 | 1 = mild<br>3 | 2 = moderate<br>4 | 3 = severe<br>5 | 4 = very severe
	161 flu_b4_cfs_1<br><br>Show the field ONLY if:<br>[me_cfs_1] = '1'	53. Flu-like symptoms: Did you have this symptom BEFORE CFS?	yesno, Required<br>1 | Yes<br>0 | No
	162 flu_freq_1<br><br>Show the field ONLY if:<br>[me_cfs_1] = '1'	Over the PAST 6 MONTHS, how OFTEN have you had this symptom?: Flu-like symptoms	radio, Required<br>1 | 0 = none of the time<br>2 | 1 = a little of the time<br>3 | 2 = about half the time<br>4 | 3 = most of the time<br>5 | 4 = all of the time
	163 flu_severe_1<br><br>Show the field ONLY if:<br>[me_cfs_1] = '1'	Over the PAST 6 MONTHS, how MUCH has this symptom bothered you?: Flu-like symptoms	radio, Required<br>1 | 0 = symptom not present<br>2 | 1 = mild<br>3 | 2 = moderate<br>4 | 3 = severe<br>5 | 4 = very severe
	164 some_smells_b4_cfs_1<br><br>Show the field ONLY if:<br>[me_cfs_1] = '1'	54. Some smells, foods, medications, or chemicals make you feel sick: Did you have this symptom BEFORE CFS?	yesno, Required<br>1 | Yes<br>0 | No
	165 some_smells_freq_1<br><br>Show the field ONLY if:<br>[me_cfs_1] = '1'	Over the PAST 6 MONTHS, how OFTEN have you had this symptom? Some smells, foods, medications, or chemicals make you feel sick	radio, Required<br>1 | 0 = none of the time<br>2 | 1 = a little of the time<br>3 | 2 = about half the time<br>4 | 3 = most of the time<br>5 | 4 = all of the time

	166	some_smells_severe_1<br><br>Show the field ONLY if:<br>[me_cfs_1] = '1'	Over the PAST 6 MONTHS, how MUCH has this symptom bothered you? Some smells, foods, medications, or chemicals make you feel sick	radio, Required<br>1 0 = symptom not present<br>2 1 = mild<br>3 2 = moderate<br>4 3 = severe<br>5 4 = very severe
	167	ic_sym_q1_1	During the PAST MONTH: How often have you felt the strong need to urinate with little or no warning?	radio, Required<br>1 Not at All<br>2 Less than 1 time in 5<br>3 Less than half the time<br>4 About half the time<br>5 More than half the time<br>6 Almost always
	168	ic_sym_q2_1	During the PAST MONTH: Have you had to urinate less than 2 hours after you finished urinating?	radio, Required<br>1 Not at All<br>2 Less than 1 time in 5<br>3 Less than half the time<br>4 About half the time<br>5 More than half the time<br>6 Almost always
	169	ic_symp_q3_1	During the PAST MONTH: How often do you most typically get up at night to urinate?	radio, Required<br>1 None<br>2 Once<br>3 2 times<br>4 3 times<br>5 4 times<br>6 5 or more times
	170	ic_symp_q4_1	During the PAST MONTH: Have you experienced pain or burning in your bladder?	radio, Required<br>1 Not at all<br>2 A few times<br>3 Almost always<br>4 Fairly often<br>5 Usually
	171	ic_prob_intro_1	During the PAST MONTH how much has each of the following been a problem for you?	descriptive
	172	ic_prob_q1_1	Frequent urination during the day?	radio, Required<br>1 No problem<br>2 Very small problem<br>3 Small problem<br>4 Medium problem<br>5 Big problem
	173	ic_prob_q2_1	Getting up at night to urinate?	radio, Required<br>1 No problem<br>2 Very small problem<br>3 Small problem<br>4 Medium problem<br>5 Big problem

174	ic_prob_q3_1	Need to urinate with little warning?	radio, Required<br>1 No problem<br>2 Very small problem<br>3 Small problem<br>4 Medium problem<br>5 Big problem
175	ic_prob_q4_1	Burning, pain, discomfort, or pressure in your bladder?	radio, Required<br>1 No problem<br>2 Very small problem<br>3 Small problem<br>4 Medium problem<br>5 Big problem
176	end_date_core_1_1	Date Questionnaire was completed	text (date_mdy), Required
177	cfs_symptom_questionnaire_complete	Section Header: *Form Status*<br>Complete?	dropdown<br>0 Incomplete<br>1 Unverified<br>2 Complete

Instrument: **SF-36** (sf36) Collapse

178	id_check1	Please re-enter the Participant ID here (for quality control purposes)	text, Required
179	rand36_1_1	1. In general, would you say your health is:	radio, Required<br>100 1, Excellent<br>75 2, Very good<br>50 3, Good<br>25 4, Fair<br>0 5, Poor
180	rand36_2_1	2. Compared to one year ago, how would your rate your health in general now?	radio, Required<br>100 1, Much better now than one year ago<br>75 2, Somewhat better now than one year ago<br>50 3, About the same<br>25 4, Somewhat worse now than one year ago<br>0 5, Much worse now than one year ago
181	ghq_instr_1	The following items are about activities you might do during a typical day. Does your health now limit you in these activities? If so, how much? (Choose one answer for each question.)	descriptive
182	rand36_3_1	3. Vigorous activities, such as running, lifting heavy objects, participating in strenuous sports	radio, Required<br>0 1, Yes, limited a lot<br>50 2, Yes, limited a little<br>100 3, No, not limited at all
183	rand36_4_1	4. Moderate activities, such as moving a table, pushing a vacuum cleaner, bowling, or playing golf	radio, Required<br>0 1, Yes, limited a lot<br>50 2, Yes, limited a little<br>100 3, No, not limited at all

	184	rand36_5_1	5. Lifting or carrying groceries	radio, Required<br>0 1, Yes, limited a lot<br>50 2, Yes, limited a little<br>100 3, No, not limited at all
	185	rand36_6_1	6. Climbing several flights of stairs	radio, Required<br>0 1, Yes, limited a lot<br>50 2, Yes, limited a little<br>100 3, No, not limited at all
	186	rand36_7_1	7. Climbing one flight of stairs	radio, Required<br>0 1, Yes, limited a lot<br>50 2, Yes, limited a little<br>100 3, No, not limited at all
	187	rand36_8_1	8. Bending, kneeling, or stooping	radio, Required<br>0 1, Yes, limited a lot<br>50 2, Yes, limited a little<br>100 3, No, not limited at all
	188	rand36_9_1	9. Walking more than a mile	radio, Required<br>0 1, Yes, limited a lot<br>50 2, Yes, limited a little<br>100 3, No, not limited at all
	189	rand36_10_1	10. Walking several blocks	radio, Required<br>0 1, Yes, limited a lot<br>50 2, Yes, limited a little<br>100 3, No, not limited at all
	190	rand36_11_1	11. Walking one block	radio, Required<br>0 1, Yes, limited a lot<br>50 2, Yes, limited a little<br>100 3, No, not limited at all
	191	rand36_12_1	12. Bathing or dressing yourself	radio, Required<br>0 1, Yes, limited a lot<br>50 2, Yes, limited a little<br>100 3, No, not limited at all
	192	ghq_instr2_1	During the past 4 weeks, have you had any of the following problems with your work or other regular daily activities as a result of your physical health? (Choose one answer for each question.)	descriptive
	193	rand36_13_1	13. Cut down the amount of time you spent on work or other activities	radio, Required<br>0 1, Yes<br>100 2, No
	194	rand36_14_1	14. Accomplished less than you would like	radio, Required<br>0 1, Yes<br>100 2, No
	195	rand36_15_1	15. Were limited in the kind of work or other activities	radio, Required<br>0 1, Yes<br>100 2, No

	196	rand36_16_1	16. Had difficulty performing the work or other activities (for example, it took extra effort)	radio, Required<br>0 | 1, Yes<br>100 | 2, No
	197	ghq_instr3_1	During the past 4 weeks, have you had any of the following problems with your work or other regular daily activities as a result of any emotional problems (such as feeling depressed or anxious)? (Choose one answer for each question.)	descriptive
	198	rand36_17_1	17. Cut down the amount of time you spent on work or other activities	radio, Required<br>0 | 1, Yes<br>100 | 2, No
	199	rand36_18_1	18. Accomplished less than you would like	radio, Required<br>0 | 1, Yes<br>100 | 2, No
	200	rand36_19_1	19. Didn't do work or other activities as carefully as usual	radio, Required<br>0 | 1, Yes<br>100 | 2, No
	201	rand36_20_1	20. During the past 4 weeks, to what extent has your physical health or emotional problems interfered with your normal social activities with family, friends, neighbors, or groups? (Choose one answer.)	radio, Required<br>100 | 1, Not at all<br>75 | 2, Slightly<br>50 | 3, Moderately<br>25 | 4, Quite a bit<br>0 | 5, Extremely
	202	rand36_21_1	21. How much bodily pain have you had during the past 4 weeks? (Choose one answer.)	radio, Required<br>100 | 1, None<br>80 | 2, Very mild<br>60 | 3, Mild<br>40 | 4, Moderate<br>20 | 5, Severe<br>0 | 6, Very severe
	203	rand36_22_1	22. During the past 4 weeks, how much did pain interfere with your normal work (including both work outside the home and housework)? (Choose one answer.)	radio, Required<br>100 | 1, Not at all<br>75 | 2, Slightly<br>50 | 3, Moderately<br>25 | 4, Quite a bit<br>0 | 5, Extremely
	204	ghq_instr4_1	These questions are about how you feel and how things have been with you during the past 4 weeks. For each question, please give the one answer that comes closest to the way you have been feeling. How much of the time during the past 4 weeks . . .(Choose one answer for each question.)	descriptive

	205	rand36_23_1	23. Did you feel full of pep?	radio, Required<br>100 | 1, All of the time<br>80 | 2, Most of the time<br>60 | 3, A good bit of the time<br>40 | 4, Some of the time<br>20 | 5, A little of the time<br>0 | 6, None of the time
	206	rand36_24_1	24. Have you been a very nervous person?	radio, Required<br>0 | 1, All of the time<br>20 | 2, Most of the time<br>40 | 3, A good bit of the time<br>60 | 4, Some of the time<br>80 | 5, A little of the time<br>100 | 6, None of the time
	207	rand36_25_1	25. Have you felt so down in the dumps that nothing could cheer you up?	radio, Required<br>0 | 1, All of the time<br>20 | 2, Most of the time<br>40 | 3, A good bit of the time<br>60 | 4, Some of the time<br>80 | 5, A little of the time<br>100 | 6, None of the time
	208	rand36_26_1	26. Have you felt calm and peaceful?	radio, Required<br>100 | 1, All of the time<br>80 | 2, Most of the time<br>60 | 3, A good bit of the time<br>40 | 4, Some of the time<br>20 | 5, A little of the time<br>0 | 6, None of the time
	209	rand36_27_1	27. Did you have a lot of energy?	radio, Required<br>100 | 1, All of the time<br>80 | 2, Most of the time<br>60 | 3, A good bit of the time<br>40 | 4, Some of the time<br>20 | 5, A little of the time<br>0 | 6, None of the time
	210	rand36_28_1	28. Have you felt downhearted and blue?	radio, Required<br>0 | 1, All of the time<br>20 | 2, Most of the time<br>40 | 3, A good bit of the time<br>60 | 4, Some of the time<br>80 | 5, A little of the time<br>100 | 6, None of the time

	211	rand36_29_1	29. Did you feel worn out?	radio, Required<br>0 — 1, All of the time<br>20 — 2, Most of the time<br>40 — 3, A good bit of the time<br>60 — 4, Some of the time<br>80 — 5, A little of the time<br>100 — 6, None of the time
	212	rand36_30_1	30. Have you been a happy person?	radio, Required<br>100 — 1, All of the time<br>80 — 2, Most of the time<br>60 — 3, A good bit of the time<br>40 — 4, Some of the time<br>20 — 5, A little of the time<br>0 — 6, None of the time
	213	rand36_31_1	31. Did you feel tired?	radio, Required<br>0 — 1, All of the time<br>20 — 2, Most of the time<br>40 — 3, A good bit of the time<br>60 — 4, Some of the time<br>80 — 5, A little of the time<br>100 — 6, None of the time
	214	rand36_32_1	32. During the past 4 weeks, how much of the time has your physical health or emotional problems interfered with your social activities (like visiting with friends, relatives, etc.)?<br><br>(Choose one number)	radio, Required<br>0 — 1, All of the time<br>20 — 2, Most of the time<br>40 — 3, A good bit of the time<br>60 — 4, Some of the time<br>80 — 5, A little of the time<br>100 — 6, None of the time
	215	ghq_instr5_1	How TRUE or FALSE is each of the following statements for you.<br><br>(Choose one answer for each question.)	descriptive
	216	rand36_33_1	33. I seem to get sick a little easier than other people	radio, Required<br>0 — 1, Definitely true<br>25 — 2, Mostly true<br>50 — 3, Don't know<br>75 — 4, Mostly false<br>100 — 5, Definitely false
	217	rand36_34_1	34. I am as healthy as anybody I know	radio, Required<br>100 — 1, Definitely true<br>75 — 2, Mostly true<br>50 — 3, Don't know<br>25 — 4, Mostly false<br>0 — 5, Definitely false

	218	rand36_35_1	35. I expect my health to get worse	radio, Required<br>0 — 1, Definitely true<br>25 — 2, Mostly true<br>50 — 3, Don't know<br>75 — 4, Mostly false<br>100 — 5, Definitely false
	219	rand36_36_1	36. My health is excellent	radio, Required<br>100 — 1, Definitely true<br>75 — 2, Mostly true<br>50 — 3, Don't know<br>25 — 4, Mostly false<br>0 — 5, Definitely false
	220	end_date_core_12_ecb	Date questionnaire was completed	text (date_mdy), Required
	221	gh_next_form	Thank you. Please indicate below when you have completed this form.<br><br>Then select "Save and go to Next Form".	descriptive
	222	sf36_complete	Section Header: *Form Status*<br>Complete?	dropdown<br>0 — Incomplete<br>1 — Unverified<br>2 — Complete
Instrument: **Multidimensional Fatigue Inventory Smets, E.M., Garseen, B., Bonke, B. & De Haes, J.C. (1995).** (multidimensional_fatigue_inventory_smets_em_garsee) Collapse				
	223	mfi_instructions_1	Below is a list of statements that describe how people sometimes feel. Please read each item carefully, then circle the one number next to each item which best describes how true each statement is about how you have been feeling lately.	descriptive
	224	mfi1_i_feel_fit_1	1. I feel fit.	radio, Required<br>0 — 1 = no, that is not true<br>16.665 — 2<br>33.332 — 3<br>49.999 — 4<br>66.666 — 5<br>83.333 — 6<br>100 — 7= yes, that is true<br><br>Custom alignment: LH

	225	mfi2_phys_do_little_1	2. Physically I feel only able to do a little.	radio, Required<br>100 — 1 = no, that is not true<br>83.333 — 2<br>66.666 — 3<br>49.999 — 4<br>33.332 — 5<br>16.665 — 6<br>0 — 7= yes, that is true<br><br>Custom alignment: LH
	226	mfi3_i_feel_active_1	3. I feel very active.	radio, Required<br>0 — 1 = no, that is not true<br>16.665 — 2<br>33.332 — 3<br>49.999 — 4<br>66.666 — 5<br>83.333 — 6<br>100 — 7 = yes, that is true<br><br>Custom alignment: LH
	227	mfi4_i_feel_nice_1	4. I feel like doing all sorts of nice things.	radio, Required<br>100 — 1 = no, that is not true<br>83.333 — 2<br>66.666 — 3<br>49.999 — 4<br>33.332 — 5<br>16.665 — 6<br>0 — 7= yes, that is true<br><br>Custom alignment: LH
	228	mfi5_i_feel_tired_1	5. I feel tired.	radio, Required<br>100 — 1 = no, that is not true<br>83.333 — 2<br>66.666 — 3<br>49.999 — 4<br>33.332 — 5<br>16.665 — 6<br>0 — 7= yes, that is true<br><br>Custom alignment: LH
	229	mfi6_i_do_a_lot_1	6. I think I do a lot in a day.	radio, Required<br>0 — 1 = no, that is not true<br>16.665 — 2<br>33.332 — 3<br>49.999 — 4<br>66.666 — 5<br>83.333 — 6<br>100 — 7 = yes, that is true<br><br>Custom alignment: LH

	230	mfi7_i_keep_thoughts_1	7. When I am doing something, I can keep my thoughts on it.	radio, Required<br>0 — 1 = no, that is not true<br>16.665 — 2<br>33.332 — 3<br>49.999 — 4<br>66.666 — 5<br>83.333 — 6<br>100 — 7 = yes, that is true<br><br>Custom alignment: LH
	231	mfi8_i_take_on_a_lot_1	8. Physically I can take on a lot.	radio, Required<br>0 — 1 = no, that is not true<br>16.665 — 2<br>33.332 — 3<br>49.999 — 4<br>66.666 — 5<br>83.333 — 6<br>100 — 7 = yes, that is true<br><br>Custom alignment: LH
	232	mfi9_dread_doing_1	9. I dread having to do things.	radio, Required<br>100 — 1 = no, that is not true<br>83.333 — 2<br>66.666 — 3<br>49.999 — 4<br>33.332 — 5<br>16.665 — 6<br>0 — 7= yes, that is true<br><br>Custom alignment: LH
	233	mfi10_i_do_little_1	10. I think I do very little in a day.	radio, Required<br>100 — 1 = no, that is not true<br>83.333 — 2<br>66.666 — 3<br>49.999 — 4<br>33.332 — 5<br>16.665 — 6<br>0 — 7= yes, that is true<br><br>Custom alignment: LH
	234	mfi11_i_concentrate_1	11. I can concentrate well.	radio, Required<br>0 — 1 = no, that is not true<br>16.665 — 2<br>33.332 — 3<br>49.999 — 4<br>66.666 — 5<br>83.333 — 6<br>100 — 7 = yes, that is true<br><br>Custom alignment: LH

	235	mfi12_im_rested_1	12. I am rested.	radio, Required<br>0 | 1 = no, that is not true<br>16.665 | 2<br>33.332 | 3<br>49.999 | 4<br>66.666 | 5<br>83.333 | 6<br>100 | 7 = yes, that is true<br><br>Custom alignment: LH
	236	mfi13_cant_concentrate_1	13. It takes a lot of effort to concentrate on things.	radio, Required<br>100 | 1 = no, that is not true<br>83.333 | 2<br>66.666 | 3<br>49.999 | 4<br>33.332 | 5<br>16.665 | 6<br>0 | 7= yes, that is true<br><br>Custom alignment: LH
	237	mfi14_phys_bad_1	14. Physically I feel I am in a bad condition.	radio, Required<br>100 | 1 = no, that is not true<br>83.333 | 2<br>66.666 | 3<br>49.999 | 4<br>33.332 | 5<br>16.665 | 6<br>0 | 7= yes, that is true<br><br>Custom alignment: LH
	238	mfi15_i_plan_1	15. I have a lot of plans.	radio, Required<br>0 | 1 = no, that is not true<br>16.665 | 2<br>33.332 | 3<br>49.999 | 4<br>66.666 | 5<br>83.333 | 6<br>100 | 7 = yes, that is true<br><br>Custom alignment: LH
	239	mfi16_i_tire_1	16. I tire easily.	radio, Required<br>100 | 1 = no, that is not true<br>83.333 | 2<br>66.666 | 3<br>49.999 | 4<br>33.332 | 5<br>16.665 | 6<br>0 | 7= yes, that is true<br><br>Custom alignment: LH

	240	mfi17_little_done_1	17. I get little done.	radio, Required<br>100 — 1 = no, that is not true<br>83.333 — 2<br>66.666 — 3<br>49.999 — 4<br>33.332 — 5<br>16.665 — 6<br>0 — 7= yes, that is true<br><br>Custom alignment: LH
	241	mfi18_dont_feel_like_doing_1	18. I don't feel like doing anything.	radio, Required<br>100 — 1 = no, that is not true<br>83.333 — 2<br>66.666 — 3<br>49.999 — 4<br>33.332 — 5<br>16.665 — 6<br>0 — 7= yes, that is true<br><br>Custom alignment: LH
	242	mfi19_thoughts_wander_1	19. My thoughts easily wander.	radio, Required<br>100 — 1 = no, that is not true<br>83.333 — 2<br>66.666 — 3<br>49.999 — 4<br>33.332 — 5<br>16.665 — 6<br>0 — 7= yes, that is true<br><br>Custom alignment: LH
	243	mfi20_phys_excellent_1	20. Physically I feel I am in an excellent condition.	radio, Required<br>0 — 1 = no, that is not true<br>16.665 — 2<br>33.332 — 3<br>49.999 — 4<br>66.666 — 5<br>83.333 — 6<br>100 — 7 = yes, that is true<br><br>Custom alignment: LH
	244	end_date_core_12_bef	Date questionnaire was completed	text (date_mdy), Required
	245	multidimensional_fatigue_inventory_smets_em_garsee_complete	Section Header: *Form Status*<br>Complete?	dropdown<br>0 — Incomplete<br>1 — Unverified<br>2 — Complete

Instrument: **Pain Inventory** (pain_inventory) Collapse

	246	everyday_pain_24_1	Throughout our lives, most of us have had pain from time to time (such as minor headaches, sprains, and toothaches). Have you had pain, other than these everyday kinds of pain during the past 24 hours?	yesno, Required<br>1 Yes<br>0 No<br><br>Custom alignment: LH
	247	widespread_pain_1	Would you consider your pain to be widespread and occurring in more than one spot on your body?	yesno, Required<br>1 Yes<br>0 No<br><br>Custom alignment: LH
	248	pain_in_24hr_1	With 0 being no pain and 10 being the worst pain you can imagine, please choose the one number that best describes your pain in the past 24 hours.	descriptive
	249	pain_at_its_worst_1	Pain at its WORST	radio, Required<br>0 0 = does not interfere<br>1 1<br>2 2<br>3 3<br>4 4<br>5 5<br>6 6<br>7 7<br>8 8<br>9 9<br>10 10 = completely interferes<br><br>Custom alignment: LH
	250	pain_at_its_least_1	Pain at its LEAST	radio, Required<br>0 0 = does not interfere<br>1 1<br>2 2<br>3 3<br>4 4<br>5 5<br>6 6<br>7 7<br>8 8<br>9 9<br>10 10 = completely interferes<br><br>Custom alignment: LH

	251	pain_on_average_1	Pain on the AVERAGE	radio, Required<br>0 | 0 = does not interfere<br>1 | 1<br>2 | 2<br>3 | 3<br>4 | 4<br>5 | 5<br>6 | 6<br>7 | 7<br>8 | 8<br>9 | 9<br>10 | 10 = completely interferes<br><br>Custom alignment: LH
	252	pain_now_1	Pain you have RIGHT NOW	radio, Required<br>0 | 0 = does not interfere<br>1 | 1<br>2 | 2<br>3 | 3<br>4 | 4<br>5 | 5<br>6 | 6<br>7 | 7<br>8 | 8<br>9 | 9<br>10 | 10 = completely interferes<br><br>Custom alignment: LH
	253	you_take_meds_1<br><br>Show the field ONLY if:<br>[please_write_treatment_1] = '' and [hr_pain_relief_1] = ''	Do you take any medications or receive any treatments for your pain?	yesno, Required<br>1 | Yes<br>0 | No<br><br>Custom alignment: LH
	254	please_write_treatment_1	Please write the treatments or medications you are taking or receiving for your pain in the box to the right.	text<br>Custom alignment: LH
	255	hr_pain_relief_1	In the past 24 hours, how much relief have pain treatments or medications provided?<br><br>0% is no relief and 100% is complete relief<br>*please do not include % sign*	text (integer, Min: 0, Max: 100), Required<br>Custom alignment: LH
	256	pain_interferes_1	With 0 being does not interfere and 10 being completely interferes, choose the one number that describes how, during the past 24 hours, pain has interfered with your:	descriptive

	257	pain_int_w_gen_activity_1	General activity	radio, Required<br>0 | 0 = does not interfere<br>1 | 1<br>2 | 2<br>3 | 3<br>4 | 4<br>5 | 5<br>6 | 6<br>7 | 7<br>8 | 8<br>9 | 9<br>10 | 10 = completely interferes<br><br>Custom alignment: LH
	258	pain_int_w_mood_1	Mood	radio, Required<br>0 | 0 = does not interfere<br>1 | 1<br>2 | 2<br>3 | 3<br>4 | 4<br>5 | 5<br>6 | 6<br>7 | 7<br>8 | 8<br>9 | 9<br>10 | 10 = completely interferes<br><br>Custom alignment: LH
	259	pain_int_w_walking_1	Walking ability	radio, Required<br>0 | 0 = does not interfere<br>1 | 1<br>2 | 2<br>3 | 3<br>4 | 4<br>5 | 5<br>6 | 6<br>7 | 7<br>8 | 8<br>9 | 9<br>10 | 10 = completely interferes<br><br>Custom alignment: LH

	260	pain_int_w_norm_work_1	Normal work	radio, Required<br>0 | 0 = does not interfere<br>1 | 1<br>2 | 2<br>3 | 3<br>4 | 4<br>5 | 5<br>6 | 6<br>7 | 7<br>8 | 8<br>9 | 9<br>10 | 10 = completely interferes<br><br>Custom alignment: LH
	261	pain_int_w_relations_1	Relations with others	radio, Required<br>0 | 0 = does not interfere<br>1 | 1<br>2 | 2<br>3 | 3<br>4 | 4<br>5 | 5<br>6 | 6<br>7 | 7<br>8 | 8<br>9 | 9<br>10 | 10 = completely interferes<br><br>Custom alignment: LH
	262	pain_int_w_sleep_1	Sleep	radio, Required<br>0 | 0 = does not interfere<br>1 | 1<br>2 | 2<br>3 | 3<br>4 | 4<br>5 | 5<br>6 | 6<br>7 | 7<br>8 | 8<br>9 | 9<br>10 | 10 = completely interferes<br><br>Custom alignment: LH

	263	pain_int_w_enjoy_life_1	Enjoyment of life	radio, Required<br>0 0 = does not interfere<br>1 1<br>2 2<br>3 3<br>4 4<br>5 5<br>6 6<br>7 7<br>8 8<br>9 9<br>10 10 = completely interferes<br><br>Custom alignment: LH
	264	end_date_core_12_625	Date questionnaire was completed	text (date_mdy), Required
	265	pain_inventory_complete	Section Header: *Form Status*<br>Complete?	dropdown<br>0 Incomplete<br>1 Unverified<br>2 Complete

Instrument: **Beck Depression Inventory-II (BDI-II). © 1996** (beck_depression_inventoryii_bdiii_copyright_1996_a)

Collapse

	266	dep_instructions_1	Check the one response to each item that best describes you for the past seven days.	descriptive
	267	bdi1_1	1) Sadness	radio, Required<br>0 I do not feel sad.<br>1 I feel sad much of the time.<br>2 I am sad all the time.<br>3 I am so sad or unhappy that I can't stand it.
	268	bdi2_1	2) Pessimism	radio, Required<br>0 I am not discouraged about my future.<br>1 I feel more discouraged about my future than I used to be.<br>2 I do not expect things to work out for me.<br>3 I feel my future is hopeless and will only get worse.
	269	bdi3_1	3) Past Failure	radio, Required<br>0 I do not feel like a failure.<br>1 I have failed more than I should have.<br>2 As I look back, I see a lot of failures.<br>3 I feel I am a total failure as a person.

	270 bdi4_1	4) Loss of Pleasure	radio, Required<br>0 I get as much pleasure as I ever did from the things I enjoy.<br>1 I don't enjoy things as much as I used to.<br>2 I get very little pleasure from the things I used to enjoy.<br>3 I can't get any pleasure from the things I used to enjoy.
	271 bdi5_1	5) Guilty Feelings	radio, Required<br>0 I don't feel particularly guilty.<br>1 I feel guilty over many things that I have done or should have done.<br>2 I feel quite guilty most of the time.<br>3 I feel guilty all of the time.
	272 bdi6_1	6) Punishment Feelings	radio, Required<br>0 I don't feel I am being punished.<br>1 I feel I may be punished.<br>2 I expect to be punished.<br>3 I feel I am being punished.
	273 bdi7_1	7) Self-Dislike	radio, Required<br>0 I feel the same about myself as ever.<br>1 I have lost confidence in myself.<br>2 I am disgusted with myself.<br>3 I dislike myself.
	274 bdi8_1	8) Self-Criticalness	radio, Required<br>0 I don't criticize or blame myself more than usual.<br>1 I am more critical of myself than I used to be.<br>2 I criticize myself for all of my faults.<br>3 I blame myself for everything bad that happe.
	275 bdi9_1	9) Suicidal Thoughts or Wishes	radio, Required<br>0 I don't have any thoughts of killing myself.<br>1 I have thoughts of killing myself, but I would not carry them out.<br>2 I would like to kill myself.<br>3 I would kill myself if I had the chance.

	276	bdi10_1	10) Crying	radio, Required<br>0 I don't cry any more than usual.<br>1 I cry more than I used to.<br>2 I cry over every little thing.<br>3 I feel like crying, but I can't.
	277	bdi11_1	11) Agitation	radio, Required<br>0 I am no more restless or wound up than usual.<br>1 I feel more restless or wound up than usual.<br>2 I am so restless or agitated that it's hard to stay still.<br>3 I am so restless or agitated that I have to keep moving or doing something.
	278	bdi12_1	12) Loss of interest	radio, Required<br>0 I have not lost interest in other people or activities.<br>1 I am less interested in other people or things than before.<br>2 I have lost most of my interest in other people or things.<br>3 It's hard to get interested in anything.
	279	bdi13_1	13) Indecisiveness	radio, Required<br>0 I make decisions about as well as ever.<br>1 I find it more difficult to make decisions than usual.<br>2 I have greater difficulty in making decisions more than I used to.<br>3 I have trouble making any decisions.
	280	bdi14_1	14) Worthlessness	radio, Required<br>0 I don't feel that I am worthless.<br>1 I don't consider myself as worthwhile and useful as I used to.<br>2 I feel more worthless as compared to other people.<br>3 I feel utterly worthless.
	281	bdi15_1	15) Loss of energy	radio, Required<br>0 I have as much energy as ever.<br>1 I have less energy than I used to have.<br>2 I don't have enough energy to do very much.<br>3 I don't have enough energy to do anything.

282	bdi16_1	16) Changes in Sleeping Pattern	radio, Required<br>0 I have not experienced any change in my sleeping pattern.<br>1 I sleep somewhat more than usual.<br>2 I sleep somewhat less than usual.<br>3 I sleep a lot more than usual.<br>4 I sleep a lot less than usual.<br>5 I sleep most of the day.<br>6 I wake up 1-2 hours early and can't get back to sleep.
283	bdi17_1	17) Irritability	radio, Required<br>0 I am no more irritable than usual.<br>1 I am more irritable than usual.<br>2 I am much more irritable than usual.<br>3 I am irritable all the time.
284	bdi18_1	18) Changes in Appetite	radio, Required<br>0 I have not experienced any change in my appetite.<br>1 My appetite is somewhat less than usual.<br>2 My appetite is somewhat greater than usual.<br>3 My appetite is much less than before.<br>4 My appetite is much greater than usual.<br>5 I have no appetite at all.<br>6 I crave food all the time.
285	bdi19_1	19) Concentration Difficulty	radio, Required<br>0 I can concentrate as well as ever.<br>1 I can't concentrate as well as usual.<br>2 It's hard to keep my mind on anything for very long.<br>3 I find I can't concentrate on anything.
286	bdi20_1	20) Tiredness or Fatigue	radio, Required<br>0 I am no more tired or fatigued than usual.<br>1 I get more tired or fatigued more easily than usual.<br>2 I am too tired or fatigued to do a lot of the things I used to do.<br>3 I am too tired or fatigued to do most of the things I used to do.

	287	bdi21_1	21) Loss of Interest in Sex	radio, Required<br>0 I have not noticed any recent change in my interest in sex.<br>1 I am less interested in sex than I used to be.<br>2 I am much less interested in sex now.<br>3 I have lost interest in sex completely.
	288	end_date_core_12_c85	Date questionnaire was completed	text (date_mdy), Required
	289	beck_depression_inventoryii_bdiii_copyright_1996_a_complete	Section Header: *Form Status*<br>Complete?	dropdown<br>0 Incomplete<br>1 Unverified<br>2 Complete

Instrument: **Beck Anxiety Inventory-II (BAI). © 1987, 1990, 1993** (beck_anxiety_inventoryii_bai_copyright_1987_1990_1) Collapse

	290	bai_instructions_1	Below is a list of common symptoms of anxiety. Please carefully read each item in the list. Indicate how much you have been bothered by that symptom during the past month, including today, by circling the number in the corresponding space in the column next to each symptom.	descriptive
	291	bai1_numbness_1	Numbness or tingling	radio, Required<br>0 Not at All<br>1 Mildly but it didn't bother me much<br>2 Moderately - it wasn't pleasant at times<br>3 Severely - it bothered me a lot
	292	bai2_feelhot_1	Feeling hot	radio, Required<br>0 Not at All<br>1 Mildly but it didn't bother me much<br>2 Moderately - it wasn't pleasant at times<br>3 Severely - it bothered me a lot
	293	bai3_wobbly_legs_1	Wobbliness in legs	radio, Required<br>0 Not at All<br>1 Mildly but it didn't bother me much<br>2 Moderately - it wasn't pleasant at times<br>3 Severely - it bothered me a lot

	294	bai4_unable_2_relax_1	Unable to relax	radio, Required<br>0 Not at All<br>1 Mildly but it didn't bother me much<br>2 Moderately - it wasn't pleasant at times<br>3 Severely - it bothered me a lot
	295	bai5_fear_of_worst_1	Fear of worst happening	radio, Required<br>0 Not at All<br>1 Mildly but it didn't bother me much<br>2 Moderately - it wasn't pleasant at times<br>3 Severely - it bothered me a lot
	296	bai6_dizzy_1	Dizzy or lightheaded	radio, Required<br>0 Not at All<br>1 Mildly but it didn't bother me much<br>2 Moderately - it wasn't pleasant at times<br>3 Severely - it bothered me a lot
	297	bai7_heart_racing_1	Heart pounding/racing	radio, Required<br>0 Not at All<br>1 Mildly but it didn't bother me much<br>2 Moderately - it wasn't pleasant at times<br>3 Severely - it bothered me a lot
	298	bai8_unsteady_1	Unsteady	radio, Required<br>0 Not at All<br>1 Mildly but it didn't bother me much<br>2 Moderately - it wasn't pleasant at times<br>3 Severely - it bothered me a lot
	299	bai9_afraid_1	Terrified or afraid	radio, Required<br>0 Not at All<br>1 Mildly but it didn't bother me much<br>2 Moderately - it wasn't pleasant at times<br>3 Severely - it bothered me a lot

	300	bai10_nervous_1	Nervous	radio, Required<br>0 Not at All<br>1 Mildly but it didn't bother me much<br>2 Moderately - it wasn't pleasant at times<br>3 Severely - it bothered me a lot
	301	bai11_choking_1	Feeling of choking	radio, Required<br>0 Not at All<br>1 Mildly but it didn't bother me much<br>2 Moderately - it wasn't pleasant at times<br>3 Severely - it bothered me a lot
	302	bai12_hands_trembling_1	Hands trembling	radio, Required<br>0 Not at All<br>1 Mildly but it didn't bother me much<br>2 Moderately - it wasn't pleasant at times<br>3 Severely - it bothered me a lot
	303	bai13_shaky_1	Shaky/unsteady	radio, Required<br>0 Not at All<br>1 Mildly but it didn't bother me much<br>2 Moderately - it wasn't pleasant at times<br>3 Severely - it bothered me a lot
	304	bai14_fear_lose_control_1	Fear of losing control	radio, Required<br>0 Not at All<br>1 Mildly but it didn't bother me much<br>2 Moderately - it wasn't pleasant at times<br>3 Severely - it bothered me a lot
	305	bai15_diff_breathing_1	Difficulty in breathing	radio, Required<br>0 Not at All<br>1 Mildly but it didn't bother me much<br>2 Moderately - it wasn't pleasant at times<br>3 Severely - it bothered me a lot

	306	bai16_fear_of_dying_1	Fear of dying	radio, Required<br>0 Not at All<br>1 Mildly but it didn't bother me much<br>2 Moderately - it wasn't pleasant at times<br>3 Severely - it bothered me a lot
	307	bai17_scared_1	Scared	radio, Required<br>0 Not at All<br>1 Mildly but it didn't bother me much<br>2 Moderately - it wasn't pleasant at times<br>3 Severely - it bothered me a lot
	308	bai18_indigest_1	Indigestion	radio, Required<br>0 Not at All<br>1 Mildly but it didn't bother me much<br>2 Moderately - it wasn't pleasant at times<br>3 Severely - it bothered me a lot
	309	bai19_faint_1	Faint/lightheaded	radio, Required<br>0 Not at All<br>1 Mildly but it didn't bother me much<br>2 Moderately - it wasn't pleasant at times<br>3 Severely - it bothered me a lot
	310	bai20_face_flush_1	Face flushed	radio, Required<br>0 Not at All<br>1 Mildly but it didn't bother me much<br>2 Moderately - it wasn't pleasant at times<br>3 Severely - it bothered me a lot
	311	bai21_hot_cold_sweat_1	Hot/cold sweats	radio, Required<br>0 Not at All<br>1 Mildly but it didn't bother me much<br>2 Moderately - it wasn't pleasant at times<br>3 Severely - it bothered me a lot
	312	end_date_part2qs_1	Date questionnaire was completed	text (date_mdy), Required

	313	beck_anxiety_inventoryii_bai_copyright_1987_1990_1_complete	Section Header: *Form Status*<br>Complete?	dropdown<br>0 Incomplete<br>1 Unverified<br>2 Complete
Instrument: **Pittsburgh Sleep Quality Index** (pittsburgh_sleep_quality_index) Collapse				
	314	psqi_instructions	The following questions relate to your usual sleep habits during the past month only. Your answers should indicate the most accurate reply for the majority of days and nights in the past month. Please answer all questions.	descriptive
	315	psqi_date_1	Date	text (date_ymd), Required
	316	psqi_time_1	Time	text (time), Required
	317	psqi_1_1	1. During the past month, what time have you usually gone to bed at night?	text (time), Required
	318	psqi_2_1	2. During the past month, how long (in minutes) has it usually taken you to fall asleep each night?	text (integer), Required
	319	psqi_3_1	3. During the past month, what time have you usually gotten up in the morning?	text, Required
	320	psqi_4_1	4. During the past month, how many hours of actual sleep did you get at night? (This may be different than the number of hours you spent in bed.)	text (number, Min: 0, Max: 24), Required
	321	psqi_instr1_1	For each of the remaining questions, check the one best response. Please answer all questions. 5. During the past month, how often have you had trouble sleeping because you . . .	descriptive
	322	psqi_5a_1	5a) Cannot get to sleep within 30 minutes	radio, Required<br>0 Not during the past month<br>1 Less than once a week<br>2 Once or twice a week<br>3 Three or more times a week
	323	psqi_5b_1	5b) Wake up in the middle of the night or early morning	radio, Required<br>0 Not during the past month<br>1 Less than once a week<br>2 Once or twice a week<br>3 Three or more times a week
	324	psqi_5c_1	5c) Have to get up to use the bathroom	radio, Required<br>0 Not during the past month<br>1 Less than once a week<br>2 Once or twice a week<br>3 Three or more times a week
	325	psqi_5d_1	5d) Cannot breathe comfortably	radio, Required<br>0 Not during the past month<br>1 Less than once a week<br>2 Once or twice a week<br>3 Three or more times a week

	326	psqi_5e_1	5e) Cough or snore loudly	radio, Required<br>0 Not during the past month<br>1 Less than once a week<br>2 Once or twice a week<br>3 Three or more times a week
	327	psqi_5f_1	5f) Feel too cold	radio, Required<br>0 Not during the past month<br>1 Less than once a week<br>2 Once or twice a week<br>3 Three or more times a week
	328	psqi_5g_1	5g) Feel too hot	radio, Required<br>0 Not during the past month<br>1 Less than once a week<br>2 Once or twice a week<br>3 Three or more times a week
	329	psqi_5j_desc_1	5j) Other Reasons (Please specify)	text
	330	psqi_5j_1	5j) Other Reasons	radio<br>0 Not during the past month<br>1 Less than once a week<br>2 Once or twice a week<br>3 Three or more times a week
	331	psqi_6_1	6. During the past month, how would you rate your sleep quality overall?	radio, Required<br>0 Very good<br>1 Fairly good<br>2 Fairly bad<br>3 Very bad
	332	psqi_7_1	7. During the past month, how often have you taken medicine to help you sleep (prescribed or "over the counter")?	radio<br>0 Not during the past month<br>1 Less than once a week<br>2 Once or twice a week<br>3 Three or more times a week
	333	psqi_8_1	8. During the past month, how often have you had trouble staying awake while driving, eating meals, or engaging in social activity?	radio, Required<br>0 Not during the past month<br>1 Less than once a week<br>2 Once or twice a week<br>3 Three or more times a week
	334	psqi_9_1	9. During the past month, how much of a problem has it been for you to keep up enough enthusiasm to get things done?	radio, Required<br>0 No problem at all<br>1 Only a very slight problem<br>2 Somewhat of a problem<br>3 A very big problem

	335	psqi_10_1	10. Do you have a bed partner or room mate?	radio, Required<br>0 No bed partner or room mate<br>1 Partner/room mate in other room<br>2 Partner in same room, but not same bed<br>3 Partner in same bed
	336	psqi_instr2_1	If you have a room mate or bed partner, ask him/her how often in the past month you have had...	descriptive
	337	psqi_10a_1<br>Show the field ONLY if:<br>[psqi_10_1] = '1' or [psqi_10_1] = '2' or [psqi_10_1] = '3'	10a) Loud snoring	radio, Required<br>0 Not during the past month<br>1 Less than once a week<br>2 Once or twice a week<br>3 Three or more times a week
	338	psqi_10b_1<br>Show the field ONLY if:<br>[psqi_10_1] = '1' or [psqi_10_1] = '2' or [psqi_10_1] = '3'	10b) Long pauses between breaths while asleep	radio, Required<br>0 Not during the past month<br>1 Less than once a week<br>2 Once or twice a week<br>3 Three or more times a week
	339	psqi_10c_1<br>Show the field ONLY if:<br>[psqi_10_1] = '1' or [psqi_10_1] = '2' or [psqi_10_1] = '3'	10c) Legs twitching or jerking while you sleep	radio, Required<br>0 Not during the past month<br>1 Less than once a week<br>2 Once or twice a week<br>3 Three or more times a week
	340	psqi_10d_1<br>Show the field ONLY if:<br>[psqi_10_1] = '1' or [psqi_10_1] = '2' or [psqi_10_1] = '3'	10d) Episodes of disorientation or confusion during sleep	radio, Required<br>0 Not during the past month<br>1 Less than once a week<br>2 Once or twice a week<br>3 Three or more times a week
	341	psqi_10e1_1<br>Show the field ONLY if:<br>[psqi_10_1] = '1' or [psqi_10_1] = '2' or [psqi_10_1] = '3'	10e) Other restlessness while you sleep<br>*Please write "N/A" if not applicable*	notes, Required
	342	end_date_core_1	Date questionnaire was completed	text (date_mdy), Required
	343	pittsburgh_sleep_quality_index_complete	Section Header: *Form Status*<br>Complete?	dropdown<br>0 Incomplete<br>1 Unverified<br>2 Complete

Instrument: **Physical Exam Checklist** (physical_exam_checklist) Collapse

	344	date_pe_ass_1	Date of Physical Exam (Assessment 1)	text (date_mdy), Required
	345	vital_signs_pe_ass_1	Vital Signs	descriptive
	346	height_pe_ass_1	Height<br>*(inches)*	text, Required
	347	weight_pe_ass_1	Weight<br>*(lbs)*	text, Required

	348	bmi_pe_ass_1	Body Mass Index (BMI)	calc, Required<br>Calculation:<br>[weight_pe_ass_1]*703/([height_pe_ass_1]*[height_pe_ass_1])
	349	temp_instru_pe_ass_1	Temperature	descriptive
	350	temp_pe_ass_1	Temperature<br>*(F)*	text, Required
	351	temp_normal_pe_ass_1	Temperature<br><br>Abnormal temperature indicates subject meets criterion Neuroendocrine-Canadian	radio, Required<br>1 Normal<br>2 Abnormal (97.0 F < Temperature > 99.6 F)
	352	temp_com_pe_ass_1	Temperature Comments	notes
	353	pain_circle_pe_ass_1	Pain (check one)	radio, Required<br>0 0 = no pain<br>1 1<br>2 2<br>3 3<br>4 4<br>5 5<br>6 6<br>7 7<br>8 8<br>9 9<br>10 10 = severe pain<br><br>Custom alignment: LH
	354	pain_normal_pe_ass_1	Pain	radio, Required<br>1 Normal (0)<br>2 Abnormal (1-10)
	355	pain_com_pe_ass_1	Pain Comments	notes
	356	cold_hands_ft_instru_pe_ass_1	Cold hands/feet	descriptive
	357	cold_hands_ft_pe_ass_1	Cold hands/feet<br><br>Cold extremities indicate subject meets criterion Neuroendocrine-Canadian	radio, Required<br>1 Normal<br>2 Abnormal
	358	cold_hands_ft_com_pe_ass_1	Cold Hands/feet Comments	notes
	359	resp_rate_pe_ass_1	Respiratory Rate	text, Required
	360	rep_rate_normal_pe_ass_1	Respiratory Rate	radio, Required<br>1 Normal<br>2 Abnormal
	361	resp_rate_com_pe_ass_1	Respiratory Rate Comments	notes

	362	bp_instru_ass_1	Blood Pressure:<br><br>1) Seated<br><br>2) Supine (record after laying down for 5 minutes)<br><br>3) Standing (record after 3 minutes)	descriptive
	363	seated_bp_pe_ass_1	Seated BP	text, Required
	364	seated_pulse_pe_ass_1	Seated Pulse	text, Required
	365	supine_bp_pe_ass_1	Supine BP	text, Required
	366	supine_pulse_pe_ass_1	Supine Pulse	text (integer), Required
	367	standing_bp_pe_ass_1	Standing BP	text, Required
	368	standing_pulse_pe_ass_1	Standing Pulse	text (integer), Required
	369	bp_normal_pe_ass_1	Blood Pressure	radio, Required<br>1 Normal<br>2 Abnormal
	370	bp_com_pe_ass_1	Blood Pressure Comments	notes
	371	pulse_normal_pe_ass_1	Pulse	radio, Required<br>1 Normal<br>2 Abnormal
	372	pulse_com_pe_ass_1	Pulse Comments	notes
	373	bp_pulse_pots_pe_ass_1<br><br>Show the field ONLY if:<br>[bp_normal_pe_ass_1] = '2' or [pulse_normal_pe_ass_1] = '2'	Signs of postural orthostatic tachycardia syndrome (POTS): drop in systolic BP > 20-25 mm of mercury upon standing AND and increase in heart rate > 30 beats per minute<br><br>POTS indicates subject meets criterion Autonomic - Canadian	yesno, Required<br>1 Yes<br>0 No
	374	bp_nmh_can_pe_ass_1<br><br>Show the field ONLY if:<br>[bp_normal_pe_ass_1] = '2'	Signs of neurally mediated hypotension (NMH): drop is systolic BP > 20-25 mm of mercury upon standing with at least 1 of the associated symptoms: lightheadedness, dizziness, visual changes, syncope, slow response to verbal stimuli, or subject feels an urgency to lie down<br><br>NMH indicates subject meets criterion Autonomic - Canadian	yesno, Required<br>1 Yes<br>0 No
	375	heent_pe_ass_1	HEENT	descriptive
	376	head_pe_ass_1	Head	radio, Required<br>1 Normal (normocephalic, normal thyroid)<br>2 Abnormal

	377	ears_pe_ass_1	Ears	radio, Required<br>1 Normal (tympanic membranes or tm's flat and no trophi)<br>2 Abnormal
	378	ears_com_pe_ass_1	Ears Comments	notes
	379	eyes_normal_pe_ass_1	Eyes	radio, Required<br>1 Normal<br>2 Abnormal
	380	eyes_pe_ass_1	Eyes<br><br>Sensitivity to light indicates subject meets criterion Neurological (Overload Phenomena) - Canadian	radio, Required<br>1 Sclerae anicteric<br>2 Sensitivity to light or diminished pupillary accommodation
	381	eyes_com_pe_ass_1	Eyes Comments	notes
	382	nose_pe_ass_1	Nose	radio, Required<br>1 Normal (pink nasal mucosa)<br>2 Abnormal
	383	nose_com_pe_ass_1	Nose Comments	notes
	384	throat_exu_pe_ass_1	Throat Exudate?	yesno, Required<br>1 Yes<br>0 No
	385	throat_ton_pe_ass_1	Throat: Tonsillar/adenoid enlargement?	yesno, Required<br>1 Yes<br>0 No
	386	throat_crim_pe_ass_1	Throat: "Crimson crescent" (anterior pharynx ring of erythema)?	yesno, Required<br>1 Yes<br>0 No
	387	throat_pe_ass_1	Throat	radio, Required<br>1 Normal (no erythema)<br>2 Abnormal
	388	throat_com_pe_ass_1	Throat Comments	notes
	389	tongue_pe_ass_1	Tongue	radio, Required<br>1 Normal (not enlarged/smooth)<br>2 Abnormal
	390	tong_com_pe_ass_1	Tongue Comments	notes
	391	skin_hair_pe_ass_1	Skin and Hair	descriptive
	392	skin_normal_pe_ass_1	Skin	radio, Required<br>1 Normal (no rash)<br>2 Abnormal
	393	skin_com_pe_ass_1	Skin Comments (comment on dry skin, folliculitis)	notes, Required

	394	hair_normal_pe_ass_1	Hair	radio, Required<br>1 Normal (no alopecia)<br>2 Abnormal
	395	hair_com_pe_ass_1	Hair Comments (comment on thinning, change in texture, etc.)	notes, Required
	396	lymph_instru_pe_ass_1	Lymph Nodes	descriptive
	397	ant_cer_normal_pe_ass_1	Anterior Cervical	checkbox, Required<br>1 ant_cer_normal_pe_ass_1___1 Normal<br>2 ant_cer_normal_pe_ass_1___2 Abnormal
	398	ant_cer_com_pe_ass_1	Anterior Cervical Comments (please comment on right/left, size, number, consistency and fixed)	notes, Required
	399	pos_cer_normal_pe_ass_1	Posterior cervical	checkbox, Required<br>1 pos_cer_normal_pe_ass_1___1 Normal<br>2 pos_cer_normal_pe_ass_1___2 Abnormal
	400	pos_cer_com_pe_ass_1	Posterior Cervical Comments (please comment on right/left, size, number, consistency and fixed)	notes, Required
	401	sub_lymph_normal_pe_ass_1	Submandibular	checkbox, Required<br>1 sub_lymph_normal_pe_ass_1___1 Normal<br>2 sub_lymph_normal_pe_ass_1___2 Abnormal
	402	sub_lymph_com_pe_ass_1_1	Submandibular Comments (please comment on right/left, size, number, consistency and fixed)	notes, Required
	403	submen_lymph_normal_pe_ass_1	Submental	checkbox, Required<br>1 submen_lymph_normal_pe_ass_1___1 Normal<br>2 submen_lymph_normal_pe_ass_1___2 Abnormal
	404	submen_lymph_com_pe_ass_1	Submental Comments (please comment on right/left, size, number, consistency and fixed)	notes, Required
	405	pre_aur_normal_pe_ass_1	Pre-auricular<br>*Always abnormal if present*	yesno, Required<br>1 Yes<br>0 No
	406	pre_aur_lymph_com_pe_ass_1	Pre-auricular (please comment on right/left, size, number, consistency and fixed)	notes, Required
	407	post_lymph_normal_pe_ass_1	Post-auricular<br>*Always abnormal if present*	yesno, Required<br>1 Yes<br>0 No
	408	post_lymph_com_pe_ass_1	Post-auricular Comments (please comment on right/left, size, number, consistency and fixed)	notes, Required
	409	occ_lymph_normal_pe_ass_1	Occipital	yesno, Required<br>1 Yes<br>0 No
	410	occ_lymph_com_pe_ass_1	Occipital Comments (please comment on right/left, size, number, consistency and fixed)	notes, Required
	411	supra_lymph_normal_pe_ass_1	Supraclavicular<br>*Always abnormal if present*	yesno, Required<br>1 Yes<br>0 No

	412	supra_lymph_com_pe_ass_1	Supraclavicular (please comment on right/left, size, number, consistency and fixed)	notes, Required
	413	ax_lymph_normal_pe_ass_1	Axillary (always abnormal if present)<br>*Always abnormal if present*	yesno, Required<br>1 Yes<br>0 No
	414	ax_com_pe_ass_1	Axillary Comments (please comment on right/left, size, number, consistency and fixed)	notes, Required
	415	epi_lymph_normal_pe_ass_1	Epitrochlear<br>*Always abnormal if present*	yesno, Required<br>1 Yes<br>0 No
	416	epi_lymph_com_pe_ass_1	Epitrochlear Comments (please comment on right/left, size, number, consistency and fixed)	notes, Required
	417	ing_lymph_normal_pe_ass_1	Inguinal<br>*Always abnormal if present*	yesno, Required<br>1 Yes<br>0 No
	418	ing_lymph_com_pe_ass_1	Inguinal Comments (please comment on right/left, size, number, consistency and fixed)	notes, Required
	419	lymph_imm_pe_ass_1<br>Show the field ONLY if: [ant_cer_normal_pe_ass_1(2)] = '1' or [pos_cer_normal_pe_ass_1(2)] = '1' or [sub_lymph_normal_pe_ass_1(2)] = '1' or [submen_lymph_normal_pe_ass_1(2)] = '1' or [pre_aur_normal_pe_ass_1] = '0' or [post_lymph_normal_pe_ass_1] = '0' or [occ_lymph_normal_pe_ass_1] = '0' or [supra_lymph_normal_pe_ass_1] = '0' or [ax_lymph_normal_pe_ass_1] = '0' or [epi_lymph_normal_pe_ass_1] = '0' or [ing_lymph_normal_pe_ass_1] = '0'	Lymphadenopathy of any region indicates subject meets criterion Immune Canadian	yesno<br>1 Yes<br>0 No
	420	pul_instru_pe_ass_1	Pulmonary	descriptive
	421	pul_normal_pe_ass_1	Pulmonary	radio, Required<br>1 Normal<br>2 Abnormal
	422	pul_com_pe_ass_1	Pulmonary Comments	notes

	423	pul_dys_def_pe_ass_1	Pulmonary Dysfunction?<br><br>Pulmonary dysfunction - irregular breathing, or holding the breath inappropriately noted after 1" of observation	yesno, Required<br>1 Yes<br>0 No
	424	pul_dys_cri_pe_1<br><br>Show the field ONLY if:<br>[pul_dys_def_pe_ass_1] = '1'	Pulmonary dysfunction indicates subject meets criterion Autonomic-Canadian	yesno, Required<br>1 Yes<br>0 No
	425	cardio_rate_pe_ass_1	Cardiac Rate	text, Required
	426	cardio_rhy_pe_ass_1	Cardiac Rhythm	radio, Required<br>1 Regular<br>2 Irregular
	427	cardio_mur_pe_ass_1	Cardiac Murmur	text, Required
	428	cardio_com_pe_ass_1	Cardiac Comments (note if history of heart attack)	notes, Required
	429	card_cri_pe_1	Rapid/Irregular heartbeat indicates subject meets criterion Autonomic - Canadian	yesno, Required<br>1 Yes<br>0 No
	430	abd_pe_ass_1	Abdominal	descriptive
	431	abd_normal_pe_ass_1	Abdominal	radio, Required<br>1 Normal<br>2 Abnormal
	432	abd_com_pe_ass_1	Abdominal Comments	notes
	433	abd_cri_pe_1<br><br>Show the field ONLY if:<br>[abd_normal_pe_ass_1] = '2'	Increased bowel sounds, mild abdominal tenderness and mild bloating indicates subject meets criterion Autonomic - Canadian	yesno, Required<br>1 Yes<br>0 No
	434	hep_pe_ass_1	Hepatomegaly	radio, Required<br>1 Normal<br>2 Abnormal
	435	hep_com_pe_ass_1	Hepatomegaly Comments	notes, Required
	436	splen_pe_ass_1	Splenomegaly	radio, Required<br>1 Normal (not larger than 2 finger breadths)<br>2 Abnormal
	437	splen_com_pe_ass_1	Splenomegaly Comments	notes
	438	splen_cri_pe_1<br><br>Show the field ONLY if:<br>[splen_pe_ass_1] = '2'	Splenomegaly indicates subject meets criterion Immune - Canadian	yesno, Required<br>1 Yes<br>0 No
	439	musc_instru_pe_ass_1	Musculoskeletal	descriptive

	440	musc_normal_pe_ass_1	Musculoskeletal	radio, Required<br>1 Normal (no tenderness)<br>2 Abnormal
	441	musc_com_pe_ass_1	Musculoskeletal Comments	notes
	442	fibro_instru_pe_ass_1	Fibromyalgia (refer to diagram with trigger points)	descriptive
	443	fm_num_pe_ass_1	FM number	text, Required
	444	fm_trig_pe_ass_1	FM location of trigger points	text
	445	fibro_com_pe_ass_1	Fibromyalgia Comments	text
	446	neuro_exam_pe_ass_1	Neurological Exam	descriptive
	447	neuro_upper_rt_pe_ass_1	Upper Extremities (Right)	text (number), Required
	448	neuro_upper_lt_pe_ass_1	Upper Extremities (Left)	text (number), Required
	449	neuro_lower_rt_pe_ass_1	Lower Extremities (Right)	text (number), Required
	450	neuro_lower_lt_pe_ass_1	Lower Extremities (Left)	text (number), Required
	451	neuro_normal_pe_ass_1	Neurologic	radio, Required<br>1 Normal<br>2 Abnormal
	452	musc_weak_pe_ass_1	Muscle weakness, twitching or ataxia?<br><br>Muscle weakness, twitching or ataxia indicates subject meets criterion Neurological (Motor Disturbance) Canadian	yesno, Required<br>1 Yes<br>0 No
	453	hyper_neuro_pe_ass_1	Hypersensitivity to vibration sense?<br><br>Hypersensitivity to vibration sense indicates subject meets criterion Neurological (Overload phenomena)-Canadian	yesno, Required<br>1 Yes<br>0 No
	454	neuro_com_pe_ass_1	Neurologic Comments	notes
	455	refl_ach_pe_ass_1	Reflexes Achilles	radio, Required<br>1 Normal<br>2 Abnormal
	456	ref_bic_pe_ass_1	Reflexes Biceps	radio, Required<br>1 Normal<br>2 Abnormal
	457	ref_pat_pe_ass_1	Reflexes Patellar	radio, Required<br>1 Normal<br>2 Abnormal
	458	ref_rel_pha_pe_ass_1	Reflexes: Attention to delayed relaxation phase<br><br>*Hypothyroidism sign*	radio, Required<br>1 Normal<br>2 Abnormal

	459	ref_com_pe_ass_1	Reflexes Comments	notes, Required
	460	gait_normal_pe_ass_1	Tandem Gait<br><br>"Abnormal" tandem gait meets Neurological (Motor disturbance) - Canadian	radio, Required<br>1 Normal<br>2 Abnormal (unable to walk heel-to-toe without corrective footing)
	461	gait_pe_com_ass_1	Tandem Gait Comments	notes, Required
	462	rho_pe_ass_1	Rhomberg<br><br>Abnormal Rhomberg indicates subject meets criterion Neurological (Motor Disturbance) - Canadian	radio, Required<br>1 Normal<br>2 Abnormal (unable to maintain balance with closed eyes and arms extended for 10 seconds without corrective footing)
	463	serial_7_pe_ass_1	Serial 7 (start at number 49)	descriptive
	464	serial_7_normal_pe_ass_1	Serial 7<br><br>Abnormal Serial 7's indicates subject meets Neurological (Impairment of Concentration) - Canadian	radio, Required<br>1 Normal<br>2 Abnormal (performed with > 2 errors)
	465	antibiotics_12_weeks	Has subject taken antibiotics since baseline?	yesno, Required<br>1 Yes<br>0 No
	466	antibiotics_12_weeks_spec	Please specify antibiotic(s) taken since baseline:	text, Required
	467	antibiotics_12_weeks_start	Please specify start date of antibiotic(s) taken since baseline:	text, Required
	468	antibiotics_12_weeks_end	Please specify end date of antibiotic(s) taken since baseline:	text, Required
	469	pe_comp_pe_ass_1	Physical Exam Completed BY:	text, Required
	470	physical_exam_checklist_complete	Section Header: *Form Status*<br>Complete?	dropdown<br>0 Incomplete<br>1 Unverified<br>2 Complete

Instrument: **Cfs Symptom Scores** (cfs_symptom_scores) Collapse

	471	interstitial_cystitis_symp_scor_1	Interstitial Cystitis Score	descriptive
	472	interstitial_cystitis_symp_1	Interstitial Cystitis Symptoms Index	calc, Identifier<br>Calculation: sum([ic_sym_q1_1],[ic_sym_q2_1],[ic_symp_q3_1],[ic_symp_q4_1])
	473	interstitial_cystitis_prob_1	Interstitial Cystitis Problem Index (ICPI)	calc<br>Calculation: sum([ic_prob_q1_1],[ic_prob_q2_1],[ic_prob_q3_1],[ic_prob_q4_1])
	474	cfs_symptom_scores_complete	Section Header: *Form Status*<br>Complete?	dropdown<br>0 Incomplete<br>1 Unverified<br>2 Complete

Instrument: **SF-36 Scores** (sf36_scores) Collapse

	475	sf_36_vitality_1	SF-36 Vitality Score	calc<br>Calculation: ([rand36_23_1]+[rand36_27_1]+[rand36_29_1]+[rand36_31_1])/4
	476	sf_36_phys_funct_1	SF-36 Physical Functioning Score	calc<br>Calculation: ([rand36_3_1]+[rand36_4_1]+[rand36_5_1]+[rand36_6_1]+[rand36_7_1]+[rand36_8_1]+[rand36_9_1]+[rand36_10_1]+[rand36_11_1]+[rand36_12_1])/10
	477	sf_36_phys_limit_1	SF-36 Physical Limitations Score	calc<br>Calculation: ([rand36_13_1]+[rand36_14_1]+[rand36_15_1]+[rand36_16_1])/4
	478	sf_36_emo_limit_1	SF-36 Emotional Limitations Score	calc<br>Calculation: ([rand36_17_1]+[rand36_18_1]+[rand36_19_1])/3
	479	sf_36_emo_wellbeing_1	SF-36 Emotional Well-Being Score	calc<br>Calculation: ([rand36_24_1]+[rand36_25_1]+[rand36_26_1]+[rand36_28_1]+[rand36_30_1])/5
	480	sf_36_soc_fuct_1	SF-36 Social Functioning Score	calc<br>Calculation: ([rand36_20_1]+[rand36_32_1])/2
	481	sf_36_pain_1	SF-36 Pain Score	calc<br>Calculation: ([rand36_21_1]+[rand36_22_1])/2
	482	sf_36_general_1	SF-36 General Score	calc<br>Calculation: ([rand36_1_1]+[rand36_33_1]+[rand36_34_1]+[rand36_35_1]+[rand36_36_1])/5
	483	sf36_scores_complete	Section Header: *Form Status*<br>Complete?	dropdown<br>0 Incomplete<br>1 Unverified<br>2 Complete
Instrument: **MFI Scores** (mfi_scores) Collapse				
	484	mfi_score_1	MFI Scores	descriptive
	485	mfi_gen_fatigue_1	MFI General Fatigue	calc<br>Calculation: ([mfi1_i_feel_fit_1]+[mfi5_i_feel_tired_1]+[mfi12_im_rested_1]+[mfi16_i_tire_1])/4
	486	mfi_phys_fatigue_1	MFI Physical Fatigue	calc<br>Calculation: ([mfi2_phys_do_little_1]+[mfi8_i_take_on_a_lot_1]+[mfi14_phys_bad_1]+[mfi20_phys_excellent_1])/4
	487	mfi_mental_fatigue_1	MFI Mental Fatigue	calc<br>Calculation: ([mfi7_i_keep_thoughts_1]+[mfi11_i_concentrate_1]+[mfi13_cant_concentrate_1]+[mfi19_thoughts_wander_1])/4
	488	mfi_red_activity_1	MFI Reduced Activity	calc<br>Calculation: ([mfi3_i_feel_active_1]+[mfi6_i_do_a_lot_1]+[mfi10_i_do_little_1]+[mfi17_little_done_1])/4

	489	mfi_red_motiv_1	MFI Reduced Motivation	calc<br>Calculation: ([mfi4_i_feel_nice_1]+[mfi9_dread_doing_1]+[mfi15_i_plan_1]+[mfi18_dont_feel_like_doing_1])/4
	490	mfi_scores_complete	Section Header: *Form Status*<br>Complete?	dropdown<br>0 Incomplete<br>1 Unverified<br>2 Complete

Instrument: **Pain Inventory Score** (pain_inventory_score) Collapse

	491	pain_interference_1	Pain Interference	calc<br>Calculation: ([pain_int_w_gen_activity_1]+[pain_int_w_mood_1]+[pain_int_w_walking_1]+[pain_int_w_norm_work_1]+[pain_int_w_relations_1]+[pain_int_w_sleep_1]+[pain_int_w_enjoy_life_1])/7
	492	pain_inventory_score_complete	Section Header: *Form Status*<br>Complete?	dropdown<br>0 Incomplete<br>1 Unverified<br>2 Complete

Instrument: **BDI Score** (bdi_score) Collapse

	493	depression_score_1	Depression Score	calc<br>Calculation: sum([bdi1_1],[bdi2_1],[bdi3_1],[bdi4_1],[bdi5_1],[bdi6_1],[bdi7_1],[bdi8_1],[bdi9_1],[bdi10_1],[bdi11_1],[bdi12_1],[bdi13_1],[bdi14_1],[bdi15_1],[bdi16_1],[bdi17_1],[bdi18_1],[bdi19_1],[bdi20_1],[bdi21_1])
	494	bdi_score_complete	Section Header: *Form Status*<br>Complete?	dropdown<br>0 Incomplete<br>1 Unverified<br>2 Complete

Instrument: **BAI Score** (bai_score) Collapse

	495	anxiety_score_1	Anxiety Score	calc<br>Calculation: sum([bai1_numbness_1],[bai2_feelhot_1],[bai3_wobbly_legs_1],[bai4_unable_2_relax_1],[bai5_fear_of_worst_1],[bai6_dizzy_1],[bai7_heart_racing_1],[bai8_unsteady_1],[bai9_afraid_1],[bai10_nervous_1],[bai11_choking_1],[bai12_hands_trembling_1],[bai13_shaky_1],[bai14_fear_lose_control_1],[bai15_diff_breathing_1],[bai16_fear_of_dying_1],[bai17_scared_1],[bai18_indigest_1],[bai19_faint_1],[bai20_face_flush_1],[bai21_hot_cold_sweat_1])

	496	bai_score_complete	Section Header: *Form Status*<br>Complete?	dropdown<br>0 Incomplete<br>1 Unverified<br>2 Complete

Instrument: **Canadian Criteria** (canadian_criteria) Collapse

	497	can_cri_pe_ass_1	Canadian Criteria	descriptive
	498	ident_cana_subject	Please choose one:	radio, Required<br>1 ME/CFS Subject<br>2 Control Subject

	499	can_cit_pe_ass_1	Please check which of the following criteria the subject meets, based on the "Physical Exam Checklist":	checkbox, Required<br>1 | can_cit_pe_ass_1___1 | Abnormal temperature (Neuroendocrine)<br>2 | can_cit_pe_ass_1___2 | Cold hands/feet (Neuroendocrine)<br>3 | can_cit_pe_ass_1___3 | POTS (Autonomic)<br>4 | can_cit_pe_ass_1___4 | NMH (Autonomic)<br>5 | can_cit_pe_ass_1___5 | Sensitivity to light (Neurological - Overload phenomena)<br>6 | can_cit_pe_ass_1___6 | Lymphadenopathy (Immune)<br>7 | can_cit_pe_ass_1___7 | Pulmonary dysfunction (Autonomic)<br>8 | can_cit_pe_ass_1___8 | Rapid/irregular heartbeat (Autonomic)<br>9 | can_cit_pe_ass_1___9 | Increased bowel sounds, mild abdominal tenderness and mild bloating (Autonomic)<br>10 | can_cit_pe_ass_1___10 | Splenomegaly (Immune)<br>11 | can_cit_pe_ass_1___11 | Muscle weakness, twitching or ataxia (Neurological - Motor disturbance)<br>12 | can_cit_pe_ass_1___12 | Hypersensitivity to vibration sense (Neurological - Overload phenomena)<br>13 | can_cit_pe_ass_1___13 | Abnormal tandem gait (Neurological - Motor disturbance)<br>14 | can_cit_pe_ass_1___14 | Abnormal Rhomberg (Neurological - Motor disturbance)<br>15 | can_cit_pe_ass_1___15 | Abnormal Serial 7's (Neurological - Impairment of concentration)
	500	canadian_criteria_complete	Section Header: *Form Status*<br>Complete?	dropdown<br>0 | Incomplete<br>1 | Unverified<br>2 | Complete

Instrument: **A1 Questions** (a1_questions) Collapse

	501	a1_ques	Has the subject withdrawn from the study?	yesno, Required<br>1 | Yes<br>0 | No

	502	a1_q_samples	Has the subject collected A1 samples?	yesno, Required<br>1 Yes<br>0 No
	503	a1_stool_coll	Date and time of A1 stool collection:	text (datetime_mdy), Required
	504	a1_stool_coll2	Date and time of second A1 stool collection (if applicable):	text (datetime_mdy)
	505	a1_sal_coll	Date and time of A1 saliva collection:	text (datetime_mdy), Required
	506	a1_sal_coll2	Date and time of second A1 saliva collection (if applicable)	text (datetime_mdy)
	507	a1_blood_coll	Date and time of A1 blood sample collection:	text (datetime_mdy), Required
	508	a1_blood_coll2	Date and time of second A1 blood sample collection (if applicable):	text (datetime_mdy)
	509	a1_questions_complete	Section Header: *Form Status*<br>Complete?	dropdown<br>0 Incomplete<br>1 Unverified<br>2 Complete
